# *DINE-1*, the highest copy number repeats in *Drosophila melanogaster* are non-autonomous endonuclease-encoding rolling-circle transposable elements (*Helentrons*)

**DOI:** 10.1186/1759-8753-5-18

**Published:** 2014-06-04

**Authors:** Jainy Thomas, Komal Vadnagara, Ellen J Pritham

**Affiliations:** 1 Department of Human Genetics, University of Utah, Salt Lake City, UT 84112, USA; 2 Department of Cancer Biology, MD Anderson Cancer Center, Houston, TX 77054, USA

**Keywords:** *Helitron*, Rolling-circle transposon, *INE-1*, *DNAREP1*, Transposable element

## Abstract

**Background:**

The Drosophila INterspersed Elements-1 (*DINE-1/INE1*) transposable elements (TEs) are the most abundant component of the *Drosophila melanogaster* genome and have been associated with functional gene duplications. *DINE-1* TEs do not encode any proteins (non-autonomous) thus are moved by autonomous partners. The identity of the autonomous partners has been a mystery. They have been allied to *Helitrons* (rolling-circle transposons), MITEs (DNA transposons), and non-LTR retrotransposons by different authors.

**Results:**

We report multiple lines of bioinformatic evidence that illustrate the relationship of *DINE-1* like TEs to endonuclease-encoding rolling-circle TEs (*Helentrons*). The structural features of *Helentrons* are described, which resemble the organization of the non-autonomous partners, but differ significantly from canonical *Helitrons*. In addition to the presence of an endonuclease domain fused to the Rep/Helicase protein, *Helentrons* have distinct structural features. Evidence is presented that illustrates that *Helentrons* are widely distributed in invertebrate, fish, and fungal genomes. We describe an intermediate family from the *Phytophthora infestans* genome that phylogenetically groups with *Helentrons* but that displays *Helitron* structure. In addition, evidence is presented that *Helentrons* can capture gene fragments in a pattern reminiscent of canonical *Helitrons*.

**Conclusions:**

We illustrate the relationship of *DINE-1* and related TE families to autonomous partners, the *Helentrons*. These findings will allow their proper classification and enable a more accurate understanding of the contribution of rolling-circle transposition to the birth of new genes, gene networks, and genome composition.

## Background

Repetitive DNA constitutes a major portion of most multicellular eukaryotic genomes. This fraction includes tandem and interspersed repeats. Transposable elements (TEs) are the major constituent of the interspersed repetitive DNA. Class 1 retrotransposons utilize an RNA intermediate and Class 2 DNA transposons utilize a DNA intermediate as the basis for transposition (for review [1]). The RNA mediated reactions are a copy-and-paste mechanism because an RNA transcript is copied to DNA. The cut-and-paste DNA transposons move the excised double-stranded DNA sequence of the transposon to a new target location (for review [1]). The rolling-circle transposons (*Helitrons*) like the retrotransposons use a copy-and-paste mechanism despite moving a DNA intermediate [2]. They are hypothesized to mobilize a single-stranded DNA molecule to a new target location [3]. Whether a TE family uses a copy-and-paste *versus* a cut-and-paste mechanism in part influences its relative abundance in a genome (for review [1]). TEs are distinguished from other forms of repetitive DNA because they replicate via self-encoded proteins (autonomous) or by hijacking TE encoded proteins (non-autonomous) (for review [4]). Hijacking the proteins of autonomous TEs appears to be a successful strategy as non-autonomous TEs generally outnumber the autonomous partners. Therefore both the replication mechanism, copy *versus* cut-and-paste and whether or not the TE is protein-coding influence copy number.

Genome-wide analysis allows the identification of entire populations of TEs in a genome, which includes old and inactive families as well as young, active copies. The classification of autonomous TEs is generally straightforward based on the encoded protein(s), the structure of the mobile unit, and the modification of the target site (TSD). While, the non-autonomous TEs do not encode proteins, they can often be classified based on structural features and TSD. Indeed, non-autonomous TE families can be linked to autonomous partners because they share common structure and TSD, which greatly facilitates classification. However, some non-autonomous families defy classification because they lack distinctive structural features, which enable a link to be made to autonomous partners.

**Figure 1:**
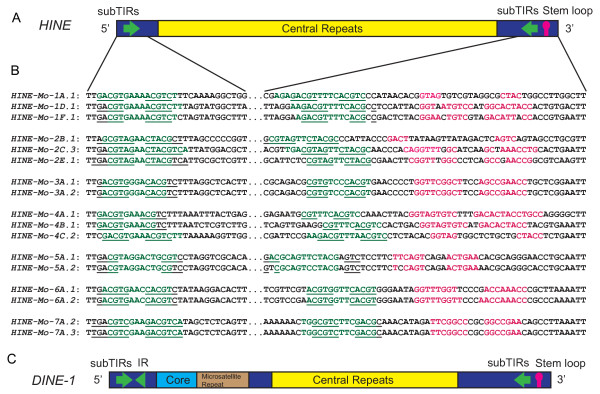
**The structural characteristics of mite *HINE *families and *Drosophila DINE-1*. (A)** The structure of the *HINE* families. The green arrows denote the subterminal inverted repeats (subTIRs). The yellow box represents the central repeats. The pink stem loop denotes the palindrome at the 3' end. **(B)** Sequence comparison of the *HINE* families and subfamilies. Mo is the abbreviation for *Metaseiulus occidentalis*, the numerals represent the family, and the subfamilies are shown by the letter number combinations. The green letters denote the subTIRs. The underlined sequence represents the palindromic sequence present internal or proximal to the subTIRs. The pink letters represent the palindrome on the 3' end. **(C)** The composite structure of *DINE-1* from the 12 Drosophila genomes (redrawn from [7]). The green arrows denote the subTIRs. The blue box represents the sequence conserved across 12 Drosophila genomes. The brown box represents the microsatellite sequence. The yellow box represents the central repeats. The green sideways triangle in the 5' end represents the 3' side of the inverted repeat (IR) (the 5' side is nested inside the 5' subTIR). The pink stem loop denotes the palindrome at the 3' end.

The Drosophila Interspersed elements (*DINE-1*) identified [5] on the dot chromosome and heterochromatic regions in the *D. melanogaster* genome fall into the category of non-autonomous TEs that have defied classification [6,7]. While *DINE-1* elements lack coding capacity they do display well-defined structural features. The structural features include 13 bp subTIRs, a short inverted repeat (IR) (approximately 3 to 22 bp away from the 5’ subTIR) and a short stem loop at the 3’ end [7] (Figure 1). Analyses of target site preferences in drosophilid genomes revealed a clear insertion preference for a TT dinucleotide but were inconclusive as far as target site modification [5-7]. These analyses allowed the definition of a core *DINE-1* sequence and the observation that they carry a short microsatellite sequence [7]. *DINE-1* elements have also been called *INE-1*[8] and DNAREP1 [9]. For the purposes of this study we refer to all related families from Drosophila as *DINE-1*.

TEs with structural characteristics similar to *DINE-1* (*DINE-1* like) have been described in the several other genomes including several drosophilids (Additional file 1: Table S1) [10-20]. *DINE-1* like elements are most abundant in the centromeres and heterochromatic regions of the chromosomes of drosophilids [5,6,10,16,21]. In *D. serido* they also predominate the heterochromatic regions of the sex chromosomes [16]. Recently active copies were found more evenly spread towards the chromosome arms and in the euchromatic regions [6,7,10]. Some *DINE-1* like elements contain tandem repeats [16,22-24] in addition to the microsatellites that are typical of the structure [7].

Outside of Drosophila, *DINE-1* like elements have been identified in many lepidopteran [25-27], hemipteran [27], and dipteran [10,28] genomes (Additional file 1: Table S1). These elements are also described from the genomes of sea urchin [24] and molluscs (Additional file 1: Table S1) [22,23,29]. *DINE-1 like* elements have attained high copy number in drosophilids [10] and other insect and invertebrate genomes (Additional file 1: Table S1) [22,24,25,27,28]. In *D. melanogaster DINE-1* elements are the most abundant TE (for review [30]) and constitute 9.2% (approximately 114 kbp) of the dot chromosome [21].

*DINE-1* like elements are reported to be involved in the duplication or generation of novel genes in Drosophila although whether this is direct or indirect is unclear and the mechanism remains elusive. Most of the known functional duplicates are associated with gametogenesis [31-34]. In *D. miranda* the *DINE-1* like elements provide the binding sites for male specific lethal complex, which regulates dosage compensation [12,35]. In addition, *DINE-1* elements are reported to provide putative transcription factor binding sites in insecticide resistance associated *Cyp* genes [36]. The myriad of ways that *DINE-1* elements have impacted Drosophila evolution [12,31-34,36], suggests many important innovations may await discovery.

The lack of structural similarity with an autonomous partner precluded the proper classification of *DINE-1* families. Indeed, these elements were initially classified as non-LTR retrotransposons perhaps because of the asymmetrical ends [5,9,11,14]. The interpretation of TSD creation led to the classification as the non-autonomous partners of classic cut-and-paste DNA transposons or Miniature Inverted-repeat Transposable Elements (MITEs) in some cases [6,10,22,23,26,27]. However, in other examples, no TSD could be readily identified and these elements were classified as *Helitrons*. Further support of the *Helitron* classification came with the identification of gene fragments with homology to the proteins encoded by some *Helitrons*, *Helentrons* and non-LTR retrotransposons [7,25,37] (for review [30]). Because the structure of the *DINE-1* like elements differed from canonical *Helitrons*[2], the relationship to *Helitrons* was not clear [7].

The canonical *Helitrons* have well defined ends (5' TC and a 3' CTRR) as well as a 16 to 20 nucleotide palindrome, which is approximately 11 bp away from the 3' end [2]. They always insert between A and T nucleotides and do not create any TSD [2]. A putatively autonomous animal *Helitron* typically encodes a single transposase open reading frame (ORF) (Rep/Helicase) with a zinc-finger, a rolling-circle motif (Rep), and a helicase domain. The plant-*Helitrons* encode additional ORFs related to ssDNA binding protein, Replication Protein A (RPA) (for review [30]). The non-autonomous partners of *Helitrons* vary in length, but share structural homology with the autonomous *Helitron* partners.

Recently two subtypes of *Helitrons* have been identified, *Helentrons*[38] and *Helitron2*[39]. *Helentrons* are so called because of the presence of an apurinic/apyrimidinic (AP) endonuclease domain fused to the C-terminus of the Rep/Helicase protein [38]. *Helentrons* encode additional ORFs with homology to OTU cysteine proteases [40,41] and RPA proteins [2]. The structural features of *Helentrons* are not known despite reports describing the coding capacity [38,40,41]. The *Helitron2* elements have asymmetrical terminal inverted repeats and palindromic sequences on both ends. These elements encode a single ORF corresponding to the Rep/Helicase protein with no endonuclease domain [39]. However, the relationship between *Helitrons*, *Helentrons*, and *Helitron2* elements is not well understood.

Here we describe for the first time the structural features of *Helentrons* and describe their relationship to *Helitrons* and *Helitron2* elements. This analysis has allowed us to unequivocally link *Helentrons* to their non-autonomous partners (the *DINE-1* like). Previously reported genomic impacts of *DINE-1* like transposons are discussed in light of the rolling-circle transposition mechanism. This includes the involvement of *DINE-1* elements in gene duplication by *de novo* chimeric gene assembly and the structural features that predispose co-option into regulatory networks. Presented is a new classification scheme for *Helentrons* and *DINE-1* like families and subfamilies that take into account their sequence heterogeneity. A model of the relationship of *Helentrons* to *Helitrons* and the intermediates identified in some organisms is presented.

## Results

### Identification and characterization of *DINE-1 like* elements in the mite genome

A TE survey of the genome of the western predatory mite, *Metaseiulus occidentalis* lead to the identification of interspersed repeat families with defined boundaries that lack coding capacity (*HINE-Mo-1-7)*. These repeats are characterized by 12 to 15 bp subterminal palindromic inverted repeats (subTIRs) and a 5 to 10 bp palindrome near the 3' end (Figure 1A-B). The subTIRs are approximately two to four nucleotides away from the 5' termini and approximately 38-60 bp away from the 3' termini. The palindromes are approximately eight to 35 bp of the 3’ termini (Figure 1A-B). Some copies contain microsatellites or tandem repeats. They display an insertion preference for T-rich sequence.

*HINE-Mo-(1-7)* repeats share most of the structural features of the *DINE-1* elements described from Drosophila, which includes the presence of subTIRs and palindromes on both ends (Figure 1C) [5-7]. Although the *HINE-Mo* subTIRs are themselves palindromic, a pattern not observed for the *DINE-1* elements (Figure 1B, C) [7]. The *HINE* families differ from *DINE-1* in that they do not have the IR and core sequence (Figure 1C) [7]. However, the common structural characteristics suggest that both *HINE* and *DINE-1* belong to the same superfamily and utilize a similar mechanism of transposition.

### Target site modifications

**Figure 2:**
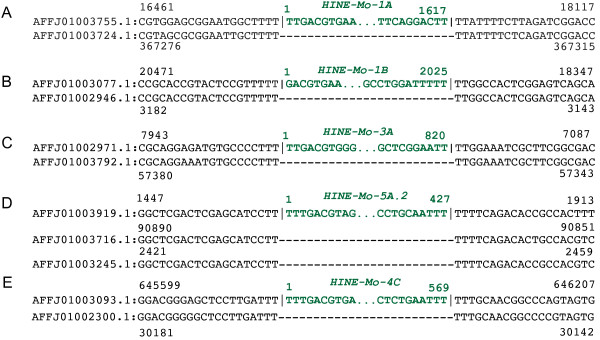
**Comparison of the host flanking sequences of individual *HINE *insertions with paralogous sites in the genome that do not have the *HINE *insertion (empty sites). (A-E)** The first line is the host sequence containing a *HINE* insertion (*HINE-Mo-1A*, *HINE-Mo-1B*, *HINE-Mo-3A*, *HINE-Mo-5A.2, HINE-Mo-4C*) in the mite genome. The second line is a paralogous site without the *HINE* insertion. The sequences in black represent the host sequence and sequence in green represents the *HINE* sequence. The accession number and coordinates are shown in black and the length of the corresponding *HINE* element is shown in green.

To confirm the boundary of *HINE-Mo* insertions, empty sites (insertion free sites) were identified (see Methods). We carefully analyzed multiple sites for each family and did not find any evidence of target site modification. *HINE* elements preferentially target a T-rich sequence and each insertion is flanked by at least two T nucleotides on each end, although it is unclear if they are distributed symmetrically (two TT on each termini) or asymmetrically (Figure 2A-C). Interestingly, in some cases elements of the same family differ in the number of T nucleotides on the termini, as evident from the analysis of paralogous empty sites (Figure 2D, E, Additional file 2: Figure S1, Additional file 3: Figure S2). Because these elements target T rich sequence, it is unclear if the Ts are distributed to only one end or both ends (Additional file 3: Figure S2). In sum, we did not find any evidence of target site modifications triggered by *HINE* integration*,* which is consistent with a single-stranded replication intermediate [3].

### Autonomous partner and the coding capacity

**Figure 3:**
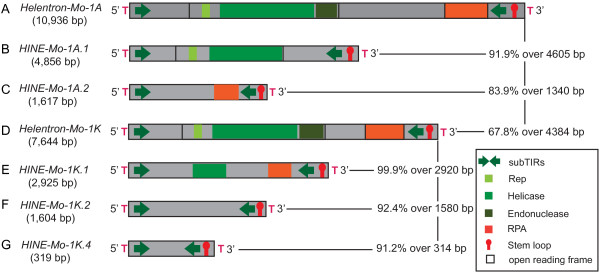
**The structural characteristics of select mite *Helentrons *and their non-autonomous partners.** The open black box denote the ORF encoding the Rep/Helicase protein (the lighter green is the Rep, the medium green is the helicase, and the endonuclease is the darker olive shade). The orange block denotes the ORF encoding the RPA protein. The green arrows represent the subterminal repeats (subTIRs). The red stem loop structure represents the 3' palindrome. The red T represents the flanking host sequence. Black vertical and horizontal lines point to the pairwise sequence identities and length of alignment (excluding gaps) of the sequences compared. **(A)** The structure of *Helentron-Mo-1A*. **(B, C)** The structures of two deletion derivatives of *Helentron-Mo-1A* (*HINE-Mo-1A.1* and *HINE-Mo-A.2*). **(D)** Is the structure of a different subfamily of *Helentrons* (*Helentron-Mo-1 K*). **(E-G)** The structures of deletion derivatives related to the *Helentron-Mo-1 K* (*HINE-Mo-1 K.1*, *HINE-Mo-1 K.2*, and *HINE-Mo-1 K.4*).

We employed homology-based searches to identify the autonomous partners of the *HINE* families. Repeats could be identified that share significant sequence identity (84% to 99.9%) with two subfamilies *HINE-Mo-1A* (Figure 3A-C) and *HINE-Mo-1 K* (Figure 3D-G). Pairwise alignments revealed that *HINE* elements are simple deletion derivatives of longer novel *Helitron-*like repeats that share the structural features displayed by the *HINEs* (Figure 3).

To determine the coding potential of the *Helitron*-like elements, we employed both conceptual translation of putative ORFs and homology-based searches (Blastx/conserved domain database (CDD)) [42] to the protein database. The CDD searches [42] and Blastx were employed to identify putative functional domains of the *Helentron* encoded proteins and related sequences in other organisms. The *Helitron*-like element from the mite encodes two putative proteins, which is atypical of animal *Helitrons* (that encode a single Rep/Helicase protein). The first corrected ORF (frame +1; no introns; 1,881 aa) shares 42% similarity over 1,336 aa with the Rep/Helicase/Endonuclease protein (Accession: DAA01284.1) identified from *Danio rerio*[38]. The alignment spanned the C-terminal endonuclease domain, which is a distinctive feature of *Helentrons* and is not associated with canonical animal *Helitrons*. The closest hit was to a putative hypothetical protein identified in the *Culex quinquefasciatus* genome (Accession: XP_001864092.1) (61% similarity over 1,747 aa).

Zn-finger-like motifs [38,40] are identified in the N terminal region of the predicted protein. The mite Rep motif shares significant sequence similarity with that of diverse *Helitrons* (*Helitron*-like, E-value: 1.62e-19, Cdd:pfam14214) [42], *Helentrons*, and the replication protein encoded by viruses and plasmids that utilize rolling-circle replication (RCR) (Additional file 4: Figure S3). The three conserved motifs that are necessary for the RCR [2,38,40,43,44] (for review [30]) are conserved in the mite *Helitron*-like element (Additional file 4: Figure S3). The helicase domain identified downstream of the Rep motif has the strongest similarity with the PIF1 family belonging to the super family 1 (SF1) of helicases (PIF1-like helicase, E-value: 6.90e-19, Cdd:pfam5970) [42]. Alignment of the eight motifs that typify the PIF1 helicase family are conserved in the both the *Helentrons* and *Helitrons* (Additional file 5: Figure S4) [2]. Fused to the C-terminus of Helicase within the same ORF, the apurinic/apyrimidinic (AP) endonuclease was identified (Exo_Endo_phos_2, E-value: 1.25e-06, Cdd:pfam14529) [42] as previously described for *Helentrons*[38]. The endonuclease of the mite *Helitron*-like element displays the characteristic seven domains described from cellular AP endonucleases, *Helentrons* and the non-LTR retrotransposon encoded protein (Additional file 6: Figure S5) [38,45].

The second corrected ORF (+3; no introns; 358 aa) putatively encodes the 70 kDa subunit of the single strand (ss) binding, Replication Protein A (RPA). The CDD searches revealed homology to the DNA binding Domains A (RPA1_DBD_A, E-value: 2.02e-15, Cdd:cd04474) and B (RPA1_DBD_B, E-value: 2.36e-12, Cdd:cd04475) [42] of RPA. Therefore the mite *Helitron*-like elements display all of the protein domains typical of *Helentrons* rather than *Helitrons*[38].

To further confirm that the mite *Helitron*-like elements belong to the *Helentron* group rather than *Helitron* group, a phylogenetic analysis (Neighbor-Joining (NJ) and maximum likelihood (ML)) was employed using an amino acid alignment of the Rep motif and eight helicase domains [46]. In this phylogenetic analysis, *Helentrons* (including the mite *Helentron*) and *Helitrons* form separate clades (*Helentron* clade bootstrap score of 95 NJ/54 ML; *Helitron* clade bootstrap score of 100 NJ/100ML) (Additional file 4: Figure S3B). Therefore the phylogenetic analysis provides an additional line of evidence that non-autonomous families from the mite genome are *Helentrons*. We call the autonomous elements, *Helentron-Mo*. Hence the non-autonomous partners are called *Helentron*-associated INterspersed Elements, in short as *HINEs*. It is interesting to note that the Rep alignment revealed diagnostic amino acid positions that in most cases can be used to quickly distinguish between proteins encoded by *Helentrons* and *Helitrons* (Additional file 4: Figure S3A). The diagnostic positions include three amino acids (F/Y w/l/y/k R) near the motif 2 ‘(V/I)ExQxRG(S/L)(P/L)HxH’ which distinguish *Helentron* protein from *Helitron* protein. In addition, the three amino acids directly preceding the well-conserved histidine residues reveal another diagnostic site. The *Helentrons* have an ‘S’ amino acid between the G and P while the *Helitrons* have an L amino acid (Additional file 4: Figure S3A). These two signatures are sufficient to distinguish *Helentrons* from *Helitrons* in our sample.

### Association of *Helentrons* and *HINEs* in other organisms

**Figure 4:**
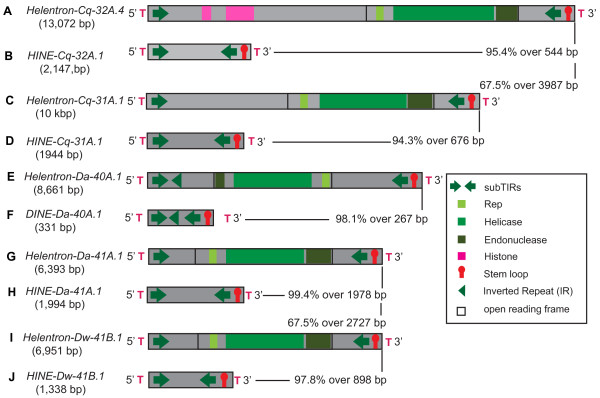
**The structural characteristics of *Helentrons *and their non-autonomous partners from other species.** The open black box denotes the ORF encoding the Rep/Helicase protein (the lighter green is the Rep, the medium green is the helicase, and the endonuclease is the darker olive shade). The orange block denotes the ORF encoding the RPA protein. The histone gene and gene fragments are represented in pink. The green arrows represent the subterminal repeats (subTIRs). The red stem loop structure represents the 3' palindrome. The green sideways triangle in the 5' end represents the 3' side of the inverted repeat (IR) (the 5' side is nested inside the 5' subTIR). The red T represents the flanking host sequence. Black vertical and horizontal lines point to the pairwise sequence identities and length of alignment (excluding gaps) of the sequences compared. **(A-D)** The structures of select *Helentrons* (*Helentron-Cq-32A.4* and *Helentron-Cq-31A.1*) and their non-autonomous partners (*HINE-Cq-32A.1* and *HINE-Cq-31A.1*) from the *Culex quinquefasciatus* genome. **(E, F)** The structure of another *Helentron* family (*Helentron-Da-40A.1*) from *D. ananassae* and the non-autonomous partner (*DINE-Da-40A.1*) described in [7]. The putative Rep/Helicase/Endonuclease in this *Helentron* is in the opposite orientation relative to others. **(G-J)** The structure of a novel family of *Helentron* (*Helentron-Da-41A.1*, *Helentron-Dw-41B.1*) present in both *Drosophila ananassae* and *D. willistoni* genomes and the non-autonomous partners (*HINE-Da-41A.1*, *HINE-Dw-41B.1*).

To determine if related *Helentrons* were present in the organisms where *DINE-1 like* families had already been described or vice versa, full-length *Helentrons* and non-autonomous families were mined from representative species (see Methods). Two families of *Helentron* and their derived *HINEs* and a novel family of *HINE* (Figure 4A-D, Additional file 7: Table S2, Additional file 8) were mined from the *C. quinquefasciatus* genome. The Culex *Helentrons* display similar structural characteristics as that of the mite including the palindromic subTIRs and the stem loop at the 3' end (Additional file 7: Table S2). The elements preferentially insert within the TT dinucleotide and have a string of Ts (2 to 5) on the boundaries (Additional file 2: Figure S1, Additional file 3: Figure S2). These Ts are part of the element but sometime vary in number between copies (Additional file 2: Figure S1, Additional file 3: Figure S2). The non-autonomous families share >95% sequence identity with the respective partner, *Helentron.* Some copies contain simple or tandem repeats that occupy approximately 50% of the total length of the element (*HINE-Cq-32A.1* and *HINE-Cq-31A.1*).

We investigated the genomes that are known to harbor recently amplified *DINE-1*s for the presence of the autonomous partner, *Helentrons* (*D. ananassae*, *D. willistoni*, and *D. yakuba*) [7]. To this end, we identified the autonomous partners of the three different *DINE-1* elements described in *D. ananassae*, *D.willistoni*, and *D. yakuba*[7] (Additional file 8). The *DINE-1*s are 98% identical with their partner *Helentron* and have similar structural characteristics including 13 bp subTIRs, IR, and a stem loop (Figure 4E, F, Additional file 7: Table S2). Interestingly, the Rep/Helicase/Endonuclease protein identified in the *Helentron-Da-40* in *D. ananassae* is in the minus orientation as opposed to plus orientation found in the majority of these elements (Figure 4E). In addition, we have identified a novel family of *Helentrons* as well as a non-autonomous family in both *D. willistoni* and *D. ananassae*. This *Helentron* family has the palindromic subTIRs instead of the IR (Figure 4G-J). As observed earlier, these elements display an insertion preference for a TT dinucleotide, have variable number of Ts at their termini, and their boundaries were confirmed by identifying paralogous empty sites (Additional file 2: Figure S1, Additional file 3: Figure S2). The copy numbers are greater than the *Helentron* partner for all the non-autonomous *Helentrons* families across different organisms. *Helentron* proteins (not full-length elements) were identified in the genomes of *Strongylocentrotus purpuratus*, *Bombyx mori*, *Danaus plexippus*, *Rhodnius prolixus*, where *DINE-1* like elements were previously reported although incorrectly annotated as MITEs [24-27] (Additional file 8). These data suggest that *Helentrons* frequently give rise to deletion derivative/non-autonomous families.

### Classification of *Helentrons* and *HINEs*

The terminal sequences including subTIRs and stem loops are shared in both *Helentrons* and *HINEs* and hence might be the signature structures necessary for the transposition. In some cases, the subTIRs are (nearly) identical (Figures 3 and 4, Additional file 7: Table S2) between two *Helentron* families that share <70% identity at the nucleotide level within a species or across species (Figures 3 and 4). Due to the heterogeneity and peculiar structural features of *Helitrons*, a new classification criterion was established to define families and subfamilies [47]. Because *Helentrons* have a different structure than *Helitrons*, the established classification scheme does not apply. To take into account the peculiar structural features of *Helentrons* in relation to *Helitrons*, we propose a new classification scheme to identify and classify different families of *Helentrons. Helentrons* containing at least 11 bp identical subTIRs are classified as members of a family (represented as the number in the name designation). Members of a subfamily share at least 80% identity over the last 60 bps of the 3' end (Figure 1) (represented as the letter following the number). The last 60 bps include the 3' subTIRs and stem loop. Using these criteria, seven families and 21 subfamilies of *Helentrons* and *HINEs* in the *M. occidentalis* genome (represented as Mo) were identified (Figure 1, Additional file 7: Table S2, Additional file 8). In addition, the mite genome harbors fragmented *Helentrons* (without ends) that diverged (>30%) from each other. This classification scheme for *Helentrons* will help to understand the diversity of these families within and across genomes.

### Distribution of *Helentrons*

We employed a homology-based search to identify autonomous *Helentrons* in the sequences available at the whole genome shotgun (wgs), Nucleotide collection (nr/nt), Genome Survey Sequences (GSS), and High Through Genome Sequence (HTGS) databases. The *Helentron* protein queries derived by the conceptual translation of ORFs encoded by mite, Culex, Drosophila, fish, and fungi were used in Tblastn searches. The conserved amino acids of Motif two of the Rep were used as a proxy to differentiate the *Helentron* from *Helitron* proteins (Additional file 4: Figure S3). These analyses reveal the presence of *Helentron* proteins in many fish, Nematostella (Cnidaria), sea urchin (Echinodermata), and insects expanding the list of those previously reported (3 more insect orders (Coleoptera, Hymenoptera, Hemiptera)); 10 more families of fish; three more families from Cnidaria and Echinodermata groups (Additional file 8) [25,38,40,41] (for review [30]) (Additional file 9: Table S3). In addition we report the presence of *Helentron* proteins in arachnids, fungi, nematodes, molluscs, rotifer, Cephalochordata, Hemichordata, Priapulida, Annelida, lampreys, and coelacanth (Additional file 8). We have identified *Helentron* proteins in a range of vertebrates and invertebrates that were not known before (Additional file 8). In addition, we identify *Helentron* proteins in the *Cotesia sesamia* Mombasa bracovirus (a virus integrated in the genome of some wasps) (for review [48]). Sequences that displayed homology to *Helentron-*Rep proteins were identified in green algae, red algae, and Oomycete genomes (Additional file 8). Interestingly, *Helentrons* are not identified in most plant genomes, but a few hits were found in the databases. Because the hits were to contigs with short length and that were low copy number, we could not rule out that they were contamination (Additional file 8). Overall, *Helentrons* have a broad distribution in Opisthokonts (for review [49]) although absent from mammals, but are otherwise very limited in their distribution taxonomically.

### Identification of a *Helitron-Helentron* intermediate

**Figure 5:**
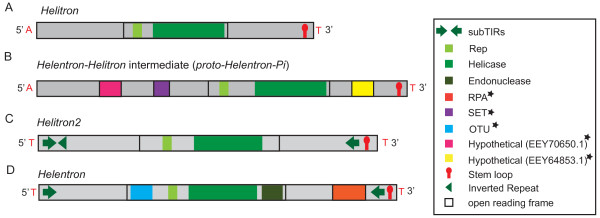
**Comparison of the structures of a typical autonomous animal *Helitron, *a potential intermediate from *Phytophthora infestans*, *Helitron2 *from *Chlamydomonas reinhardtii*, and an autonomous *Helentron *family from *Nematostella vectensis*. (A)** The structure of a representative autonomous animal *Helitron*. **(B)** The structure of the putative *Helentron* intermediate identified from the *Phyotophthora infestans* genome. **(C)** The structure of a *Helitron*2 family (redrawn from [39]). **(D)** The structure of an autonomous *Helentron*. The colored boxes represent the different encoded putative proteins or domains. The open black box denotes the ORF encoding the Rep/Helicase protein (the lighter green is the Rep, the medium green is the helicase, and the endonuclease is the darker olive shade). The orange block denotes the ORF encoding the RPA protein. The purple box represents the SET domain, blue box represents the OTU-like cysteine proteases, pink and yellow boxes represent the hypothetical proteins. Proteins and/or domains are included only if they were found in multiple families or across species. The proteins that are occasionally carried by *Helentrons* are indicated with a black asterisk (*). The green arrows denote the subterminal inverted repeats (subTIRs). The green sideways triangle in the 5' end represents the 3' side of the inverted repeat (IR) (the 5' side is nested inside the 5' subTIR). The red stem loop denotes the palindrome at the 3' end. The flanking nucleotides are shown in red.

In our survey of the *Phytophthora infestans* (Oomycete) genome, a Rep/Helicase protein (1,783 aa; +2, no introns) was identified that grouped phylogenetically with the *Helentron* Rep/Helicase group but did not have the endonuclease domain in the C terminus (Figure 5B). They have the signature amino acids in the Rep motif as observed with other *Helentrons* (Additional file 4: Figure S3). The structure of a full-length representative *Helentron* from *P. infestans*, which we call as *proto-Helentron-Pi* (approximately 14 kb) displayed the structural features more similar to *Helitrons* than *Helentrons* (5'TT and 3' CTAG) (Figure 5). The boundary of the element was confirmed by identifying a paralogous empty site (Additional file 2: Figure S1). None of the insertions are flanked by target site duplication (Figure 5, Additional file 2: Figure S1) and they insert between an A and T nucleotides like *Helitrons* (Additional file 2: Figure S1). Thus the Rep/Helicase protein is closest in amino acid similarity to *Helentrons*, yet the structural features of the complete elements are those of typical *Helitrons* (Figure 5). This suggests that *Phyotophthora proto-Helentron* could be an intermediate between *Helitron* and *Helentron* representing the element before the gain of the endonuclease domain.

The *proto-Helentron-Pi* encodes multiple putative proteins in addition to the Rep/Helicase protein. The first and last ORFs encode hypothetical proteins (+2; 218 aa; EEY70650.1 and -3; 266 aa; EEY64853.1). The second corrected ORF (approximately 153 aa, +3; no introns) encodes a putative SET domain (SET, E-value: 3.42e-24, Cdd:smart00317) [42] protein that has 50% similarity with the SET domain (121 aa) of histone H3 methyltransferase Clr4 of yeast, *Schizosaccharomyces pombe* (Accession: NP_595186.1). The characteristic features of the SET domain including the presence of variable insert region (SET-I) between the SET-N and SET-C regions (for review [50]) are present (Additional file 10: Figure S6). The highly conserved regions along with the well-conserved co-factor binding sites are present within the N and C regions (Additional file 10: Figure S6).

### Gene capture by *Helentrons*

To determine if *Helentrons* capture gene fragments like *Helitrons*, we employed Blastx/CDD searches to identify any potential gene fragment within *Helentrons* and *HINEs*. Interestingly, *Helentron-Cq-32A* in the *C. quinquefasciatus* genome carries fragments of two histone genes (H4 superfamily E-value: 1.02e-06, Cdd:smart00428 and H15 superfamily E-value: 6.34e-05, Cdd:cd00073) [42]. Eight copies carrying these gene fragments are present in the genome (Figure 4A). The H4 gene fragment within *Helentron* has 84% sequence identity over 244 bp with the putative parental in the Culex genome (AAWU01017064.1|:c10903-11310). Similarly, the H15 gene fragment has 83% identity over 87 bp with the putative parental in the Culex genome (AAWU01027066.1|:c14114-14203). However, both translated gene fragments contain a premature stop codon, frame shift, and indels when compared to the parental histone genes. The histone gene capture by a *Helentron* has not been documented in other organisms. In addition, the *P. infestans proto-Helentron* contains a fragment of a gene encoding a putative transmembrane protein (PITG_05761). The gene fragment within *Helentron* has 76% identity (1688 bp) with the putative parental gene in *P. infestans*. A short ORF (202 aa) is identified within the gene fragment in the *Helentron*, which contains the Ankyrin repeats (ANK, E-value: 5.20e-03, Cdd:cd00204) and the bacterial Toll-like Receptors (TIR_2) domain (TIR_2, E-value: 4.15e-15, Cdd: pfam13676) [42]. The *proto-Helentron-Pi* is amplified to 25 copies in the *P. infestans* genome. We did not find any potential protein-coding gene fragment within the diverse mite *Helentrons* and *HINEs* (seven families and 21 subfamilies). However, these findings suggest that *Helentrons* can transduce and amplify gene fragments but the frequency of capture varies between genomes, like their *Helitron* relatives.

## Discussion

### *Helentrons* are the autonomous partners of *DINE-1* and related TEs

Our discovery of multiple autonomous *Helentron* families in the genome of the western predatory mite facilitated the identification of features that had not been previously defined but that are common to all endonuclease-encoding *Helentrons.* The structural features and target preference for T-rich sequence are shared with many non-autonomous TE families. These families known as *DINE-1* like or *HINEs* are present in many genomes. Previously *DINE-1* like elements have been misclassified as canonical *Helitrons*, MITEs, and non-LTR retrotransposons*.* Here we solve the mystery of *DINE-1* classification by providing evidence that *Helentrons* are the autonomous partners (see Figures 3 and 4). Links were made between non-autonomous families and *Helentrons* in diverse genomes including mite, Culex, and *D. ananassae*, *D. willistoni*, and *D. yakuba*. Collectively, we call all non-autonomous *Helentrons HINEs* (for *Helentron*-associated interspersed elements).

### *HINE* genomic impact linked to rolling-circle transposition mechanism

Movement by rolling-circle transposition may help explain some of the observations previously reported for *HINE* families, for example, high copy number in some genomes, the formation of clusters, and the association with gene duplicates. *Helitrons* make up a considerable fraction of the genomes of the vespertilionid bats [51], including the little brown bat (*Myotis lucifugus)* (Thomas *et al.* in revision GBE) and the lepidopteran, *Heliconius melpomene* genome (6% and 6.62%, respectively) [52]. High genome content is consistent with a copy-and-paste transposition mechanism as is proposed for rolling-circle transposition. Clusters of *Helitrons* arranged in tandem arrays occur in the *M. lucifugus* genome [53] and *B. mori*[54] and are thought to occur when a termination signal is bypassed. In the bat and maize genome, distantly related *Helitrons* are also found in close proximity to other *Helitron* insertions sometimes directly next to one another (Thomas *et al.* in revision GBE), [47]. A similar pattern might be seen with *Helentron* mediated transposition events and might account for the clustering of related insertions [5,16,21,22,55]. Similarly, missed termination sequences or composite transposition between neighboring *Helitrons* can lead to the capture of host sequence (gene transduction). In both the bat (Thomas *et al.* in revision GBE) and maize (for example, [47]) genomes, thousands of non-autonomous elements carrying gene fragments have been described. Therefore, it is likely that the *HINEs* might also capture host sequences at some rate, *Helentrons* carry multiple additional functional genes that were likely captured. Indeed, many functional gene duplications have been described in association with *DINE-1* elements [31-34]. It may be that the gene duplications are the result of end bypass or composite rolling-circle transposition.

### Captured genes useful to *Helentron* lifecycle

The Rep/Helicase protein encoded by *Helentrons* has an endonuclease domain fused to the C-terminus. This endonuclease is most closely related to the proteins encoded by CR1 non-LTR retrotransposons [38,41] and was either captured when a non-LTR retrotransposed into a *Helitron* or via a DNA-based transduction event (Thomas *et al.* in revision GBE). For CR1 retrotransposons target preference is likely determined by the sequence at the 3' end of the element [56] and is thought to be guided by the endonuclease as has been shown for other non-LTR retrotransposons [45,57-59]. In target primed reverse transcription, the endonuclease creates the single stranded nick at the target site to initiate the target primed reverse transcription [45,60]. We hypothesize that the target preference for the poly T track of the *Helentrons* is guided by the sequence (poly T or A on complementary strand) at the termini of *Helentron*. As observed with non-LTRs, the endonuclease may be playing a role in target preference and might be generating the single-stranded nick necessary for transposition initiation. We hypothesize that the initial nick in the T sequence flanking the element results in the variability of T nucleotides flanking the elements. Biochemical studies will be necessary to illuminate the mechanism of transposition of these elements.

We find no evidence that either *Helentrons* or *HINEs* create target site modification as has sometimes been reported in previous studies [6,7,16,22,23,26,27]. Analysis of the target site is made complicated by target preference for poly T sequences and the presence of an unknown and variable number of T nucleotides flanking individual elements (Figure 2, Additional file 2: Figure S1, Additional file 3: Figure S2). We propose that the variable number of T nucleotides at the termini of the element is the result of the endonuclease indiscriminately cleaving between T dinucleotides in the poly T stretch flanking the element for the initial single stranded cleavage.

The conservation and the probable utilization of the endonuclease for function illustrates that the capture of host genes can lead to innovation in transposition mechanism, which might have downstream consequences to the structure of the elements. Indeed, we identified an intermediate *Helentron* family that did not encode the endonuclease from Phytophthora (Figure 5). The Rep/Helicase encoded by this family group phylogenetically with the proteins from *Helentrons* (Additional file 4: Figure S3). However, the element has the structural features of a canonical *Helitron* and integration occurs between A and T nucleotides.

It is possible that other captured genes (Figure 5, Additional file 9: Table S3) could aid transposition. It has been proposed that the RPA encoded proteins independently captured by *Helitrons* in plants and *Helentrons* might bind single stranded DNA during transposition [2], (for review [30]). A role of the OTU-like cysteine proteases in transposition is supported by the conservation of all residues required for function and the presence of the genes in *Helentron* families from diverse species [40]. The protease might be involved in inducing proteolytic events involved in signaling necessary to trigger the modification of chromatin structure [61,62] or in cleaving the Rep/Helicase/Endonuclease subunits (for review [30]), which might facilitate transposition. Further experimental studies are necessary to understand mechanisms involved in transposition.

### Gene fragment transduction and duplication by *Helentrons*

We looked for evidence for gene captures using homology based methods in the set of autonomous and non-autonomous elements identified in our study. Besides previously annotated examples, we only identified gene fragments in the *Helentrons* from the Culex and Phytophthora genomes (Additional file 9: Table S3). We did not detect gene fragments within *Helentrons* or *HINEs* as with the frequency observed in maize or bat *Helitrons* (>10,000 *Helitrons* containing gene fragments) (Thomas *et al.* in revision GBE) (for example, [47]. A caveat to this approach is the reliance on significant homology with known proteins. It may be that the frequency of gene captures simply varies with certain genomes, as has been observed with *Helitrons*[63].

Previous studies have identified non-random associations of *HINE*-like elements at or near the break points of several functional gene duplicates in Drosophilids [31-34]. In some of these cases, duplications occurred before as well as after speciation events. For example, for six *Kep 1* gene fragment duplications, four duplications occurred before the diversification *D. melanogaster* species complex (that is, 2-3 to 6-7 million years ago) [34] while one occurred in *D. melanogaster* and one occurred in an ancestor of *D. sechellia* and *D. simulans*[34]. If the *Kep 1* gene fragment is carried by a *HINE*-like element and duplication occurred as the result of transposition, this pattern would suggest that *Helentrons* were active throughout the diversification of the *D. melanogaster* species complex. Other possible gene transduction events include *CK2βtes* and *NACβtes* gene duplicates also from the *D. melanogaster* species complex [33]. The *NACβtes* copies were amplified before the diversification of *D. melangaster* species complex and *CK2βtes* gene duplicates have amplified in the *D. sechellia*/*D. simulans* lineage [33]. The *hydra* gene was uniquely amplified after the insertion of a *HINE* in the *D. melanogaster* species complex [31]. A burst of *HINE* transposition occurred in the ancestor of *D. melanogaster* species complex and it has been suggested that activity may have continued after diversification [64]. These duplication events do correlate well with the peak of *HINE* activity (approximately 4.6 million years ago) (average divergence 15.2% ±5.4 SD) [64]. Pairwise divergence (1% to 19%) estimates of the *DINE* copies in the *D. sechellia* and *D. simulans* suggest a longer potential period of activity as compared to *D. melanogaster* (3-19%) [7]. *Helitrons* in bats have maintained activity for approximately 36 million years (Thomas *et al.* in revision GBE). These findings indeed suggest that *Helentron* activity could play an important role in the amplification and dispersal of genic fragments and the generation of new functional gene duplicates. It maybe that *Helentrons* like *Helitrons* are capable of long periods of activity [12,64].

## Conclusions

In this study, we characterized the structural features of *Helentrons* and identify that they are different from the typical structural features of canonical *Helitrons*. In addition, we found that *Helentrons* and *DINE-1* like elements share similar structural features, which unequivocally links the *DINE-1* like elements as non-autonomous partners of *Helentrons. Helentrons* and its non-autonomous partners do not induce target site duplications upon transposition, but are flanked by variable number of Ts. We have also identified potential intermediates that have Rep/Helicase protein similar to *Helentrons* but share the structural characters with *Helitrons*. Hence our study provides a better understanding of the structure and distribution of *Helitron*-like elements across taxa. In addition, our studies illustrate that *Helentrons* are capable of gene transduction as their *Helitron* relatives.

## Methods

### Identification of *HINEs* and *Helentrons* from the mite genome and other selected genomes

*Helentrons* and *HINEs* were identified from the genome of the mite, *Metaseiulus occidentalis* during a *de novo* analysis of repeats. Repeatscout [65] was used to generate a consensus of the repeats present in three or more copies in the genome and *HINEs* were identified during the manual curation of these repeats. The structural characteristics of the *HINE* elements and *Helentrons* were compared and analyzed using Blast tools [66]. To identify the distribution pattern, Tblastn searches were carried out using the mite *Helentron* protein query against wgs, nr, GSS, and HTGS databases. Full-length *Helentrons* and *HINE*s were mined from Culex, *D. willistoni*, *D. ananassae*, and *D. yakuba* genomes to verify the relationship between *Helentron* and *HINEs*.

### Identification of open reading frames, conserved domains, and gene fragments

The Translate (http://web.expasy.org/translate/) and ORF finder (http://www.ncbi.nlm.nih.gov/gorf/gorf.html) tools were utilized to identify *Helentron* encoded ORFs. The stop codons and frame shift mutations were corrected to obtain an intact the ORF, if necessary. The conserved domain database searches (CDD) [42] and Blastx were employed to identify putative functional domains of the *Helentron* encoded proteins and related sequences in other organisms. The low complexity filter was applied during CDD searches to avoid spurious results. Significant hits (e-values <10^-03^) were further explored. To identify potential protein-coding gene fragments within *Helentrons* and *HINEs*, we employed Blastx/CDD based searches and significant (e-values <10^-03^) hits (other than TEs) were further explored.

### Identification of paralogous empty sites

To confirm the boundary of the elements, paralogous sites without the insertion (empty sites) were identified. To identify empty sites, a chimeric query constructed from 50 bp upstream and downstream of the element was utilized for homology-based searches (Blastn) against genomes. Hits with ≥90% identity over 90% of the query are considered as an empty site.

### Alignments and phylogenetic analysis

Alignment of the putative proteins encoded by *Helentrons*, *Helitrons*, and other bacterial plasmids or viruses that encode similar proteins were constructed employing MUSCLE (http://www.ebi.ac.uk/Tools/msa/muscle/) using default parameters. The alignments were visually refined using Genedoc (v. 2.7), [67]. Phylogenetic analysis of the Rep motifs and helicase domains was conducted using MEGA (v 5.05) [46] by constructing a NJ tree and the parameters selected were JTT matrix based model, 1,000 bootstrap replicates and pairwise deletion. A maximum likelihood analysis (JTT matrix based model, 1,000 bootstrap replicates) was also performed using MEGA (v 5.05) [46].

## Abbreviations

CDD: Conserved domain database; DINE: Drosophila interspersed element; HINE: Helentron associated interspersed element; JTT: Jones Taylor Thornton; LTR: Long terminal repeat; MITEs: Miniature inverted terminal repeat elements; ORF: Open reading frame; OTU: Ovarian tumor; RPA: Replication Protein A; SD: Standard deviation; SET: Su(var)3-9 and ‘Enhancer of zeste’; TE: Transposable element; TIR: Toll-like Receptors; TSD: Target site duplication.

## Competing interests

The authors declare that they have no competing interests.

## Authors’ contributions

JT conceived, designed, and conducted the study, and drafted the manuscript. KV participated in the study by mining the *Phytophthora infestans Helentron* and describing the coding capacity. EJP conceived of the study, participated in the design, and drafted the manuscript. All authors read and approved the final manuscript.

## Supplementary Material

Additional file 1: Table S1 Distribution and copy number of *DINE-1*-like elements. Click here for file

Additional file 2: Figure S1 Comparisons of the host flanking sequences of individual *HINE* insertions with paralogous sites in the genome that do not have the *HINE* insertion (empty sites). The first line is the host sequences with the *Helentrons/HINE* insertion. The second line is a paralogous site without the *Helentron/HINE* insertion. The black nucleotides represent the host sequence and underlined red nucleotides represent the transposable element. The accession and coordinates of the sequences are also given in black and the length of the transposable element is shown in red. **(A-E)** Empty sites of select *Helentron/HINE* insertions in *Drosophila ananassae* (*HINE-Da-41A.2*), *D. willistoni* (*Helentron-Dw-41B.1*), *Culex quinquefasciatus* (*HINE-Cq-32A.1*, *HINE-Cq-32A.2*), and *Phytophthora infestans* (*proto-Helentron-Pi*). Click here for file

Additional file 3: Figure S2 Comparison of the host flanking sequences of multiple *HINE* insertions and insertion free sites (empty sites) in the genome. The underlined sequences in red represent the *HINEs* and black nucleotide represent the host sequence. The accession and coordinates of the sequences are also given in black. **(A)** Multiple *HINE-Da-41A* insertions with their flanking sequences in the *Drosophila ananassae* genome. **(B)** Empty sites for each *HINE-Da-41A* insertion. The first line is the host sequence with the *HINE-Da-41A* insertion. The second line is a paralogous site without the *HINE* insertion. **(C)** Multiple *HINE-Mo-4C* insertions with flanking sequences in the *Metaseiulus occidentalis* genome. **(D)**. Empty sites for each *HINE-Mo-4C* insertion. The first line is the host sequence with the *HINE-Mo-4C* insertion. The second line is a paralogous site in the genome without the *HINE* insertion. Click here for file

Additional file 4: Figure S3 The protein alignment of the Rep motif of representative *Helentrons* and *Helitrons* and a phylogenetic tree based on an alignment of the most conserved Rep motifs/Helicase domains. **(A)** An alignment of the Rep motif of *Helentrons* from 12 species, *Helitrons* from seven species and representative plasmids and viruses that utilizes rolling-circle replication (RCR). Black asterisks above the alignment denote the positions of the two histidines and two tyrosines known to be critical for catalytic activity of the RC elements. Identical residues are shaded in black and conservative changes are shaded in gray. Amino acids that distinguish *Helentrons* from *Helitrons* are boxed in red. The accession and coordinates of the different sequences used in the alignment are: *Helentrons* from *Metaseiulus occidentalis* Mite-1 (AFFJ01001714.1:c5449-8790), Mite-3 (AFFJ01002321.1:c999-4343) *Culex quinquefasciatus* (AAWU01024641.1:12176- 15496), platyfish *Xiphophorus maculatus* (ABB05534.1), fungi *Mucor circinelloides* (EPB86818.1), acornworm *Saccoglossus kowalevskii* (XP_002741052.1), *Phytophthora infestans* (AATU01002056.1:10099-12180), *Nematostella vectensis* (*Helitron-1_NV*) [30], sea urchin *Strongylocentrotus purpuratus* (AAGJ04076666.1:8326-11865), *Danio rerio* (DAA01284.1), Frog *Xenopus tropicalis* (AAMC02019010.1: 25350- 33598) *Drosophila willistoni* (AAQB01006357.1:146323-152490), *D. ananassae* (AAPP01019845.1:107830-112664), *D. yakuba* (AAEU02001960.1:c3447-10117). *Helitrons* from mite *M. occidentalis* (AFFJ01001759.1:1748-4869), *D. ananassae* (AAPP01018364.1:33765-39124), Aphid *Acyrthosiphon pisum* (AC202211.4:97955-103017), *Myotis lucifugus* (AAPE02018439.1:1503-5146), *Bombyx mori Helianu_Bm1*[54], *Oryza sativa japonica* (AAM92800.1), and *Arabidopsis thaliana* (AtHEL2p) [2]. SVTS, *Spiroplasma plectro* virus (AAF18311.2); Rep_SC, *Streptomyces cyaneus* plasmid (BAA34784.1); Rep_BB, *Bacillus borstelensis* plasmid (BAA07788.1); Rep_AA, *Actinobacillus actinomycetemcomitans* plasmid (AAC37125.1); Pf3, *Pseudomonas aeruginosa* bacteriophage (AAA88392). **(B)** A Neighbor-Joining (NJ) tree generated from the Rep motif and helicase domains. The bootstrap values calculated from maximum likelihood are listed before the backslash at each node and followed by bootstrap values calculated as part of the NJ analysis. The symbols in the tree are explained in the boxed legend. Click here for file

Additional file 5: Figure S4 Protein alignment of the PIF1 helicase from *Helentrons*, *Helitrons*, and select organisms. The eight conserved motifs of the PIF1 family of helicases from *Helentrons*, *Helitrons*, yeast (P07271), baculovirus (Q9YMS4), TRAA_RHISN, *Rhizobium* sp (P55418.1), (P55418.1), and T4 phage (P32270). The accession and coordinates of the *Helentrons* and *Helitrons* used in the alignment are: *Helentrons* from *Metaseiulus occidentalis* Mite-1 (AFFJ01001714.1:c5449-8790), Mite-3 (AFFJ01002321.1:c999-4343) *Culex quinquefasciatus* (AAWU01024641.1:12176-15496), platyfish *Xiphophorus maculatus* (ABB05534.1), fungi *Mucor circinelloides* (EPB86818.1), acornworm *Saccoglossus kowalevskii* (XP_002741052.1), *Phytophthora infestans* (AATU01002161.1: 20532-18847), *Nematostella vectensis* (*Helitron-1_NV*) [30], sea urchin *Strongylocentrotus purpuratus* (AAGJ04076666.1:8326-11865), *Danio rerio* (DAA01284.1), Frog *Xenopus tropicalis* (AAMC02019010.1: 25350- 33598), *Drosophila willistoni* (AAQB01006357.1:146323-152490), *D. ananassae* (AAPP01019845.1:107830-112664), *D. yakuba* (AAEU02001960.1:c3447-10117). *Helitrons* from Mite *M. occidentalis* (AFFJ01001759.1:1748-4869), *D. ananassae* (AAPP01018364.1:33765-39124), Aphid *Acyrthosiphon pisum* (AC202211.4:97955-103017), *Rhodnius prolixus HeligloriaAi_Rp1* (ACPB01050589.1:10663-14351), *Bombyx mori Helianu_Bm1*[54], *Oryza sativa japonica* (AAM92800.1), and *Arabidopsis thaliana* (AtHEL2p) [2]. Click here for file

Additional file 6: Figure S5 An alignment of the apurinic/apyrimidinic endonuclease alignment encoded by *Helentrons*, non-LTR retrotransposons and select cellular proteins. A protein alignment of the endonuclease domains of *Helentrons* from 12 species, non-LTR retrotransposons from five species, and three cellular endonucleases. The accession and coordinates of the different sequences used in the alignment are: *Helentrons* from *Metaseiulus occidentalis* Mite-1 (AFFJ01001714.1:c5449-8790), Mite-2 (AFFJ01002369.1:4460-5251) *Culex quinquefasciatus* (AAWU01024641.1:12176- 15496), platyfish *Xiphophorus maculatus* (ABB05534.1), fungi *Mucor circinelloides* (EPB86818.1), acornworm *Saccoglossus kowalevskii* (XP_002741052.1), sea urchin *Strongylocentrotus purpuratus* (AAGJ04076666.1:8326-11865), *Danio rerio* (DAA01284.1), Frog *Xenopus tropicalis* (AAMC02019010.1: 25350-33598), *Drosophila willistoni* (AAQB01006357.1:146323-152490), *D. ananassae* (AAPP01019845.1:107830-112664), *D. yakuba* (AAEU02001960.1:c3447-10117). The cellular endonucleases are from *Bos taurus* APEX1_BOVIN (P23196.2), *Homo sapiens* APEX (AAB26054.1), *Escherichia coli* APEX3 (AAC74819.1). The non-LTR are from *Daphnia pulex* (EFX61861.1), *Trypanosoma cruzi* (CAB41692.1), *Danio rerio* (BAE46430.1), *Oryzias latipes* ReO_6 (BAB83841.1), *Nematostella vectensis* -Rex1_CR1, and *C.elegans*-Frodo_CR1 [38]. Click here for file

Additional file 7: Table S2 The subterminal inverted repeats (subTIRs) of *Helentron-HINE* families identified from different species. Click here for file

Additional file 8: File S1 Accession number and coordinates of full length *Helentrons* and *HINEs* identified in the *Metaseiulus occidentalis*, *Culex quinquefasciatus*, Drosophila, Phytophthora genomes and *Helentron* proteins identified in different organisms for which the sequences are deposited in whole genome shotgun (wgs), Genome Sequence Survey (GSS), High Throughput Genome Sequence (HTGS) databases, and Nucleotide collection (nr/nt) databases. Click here for file

Additional file 9: Table S3 The presence of genes in *Helentron* families identified in this study or previously described in the literature. Click here for file

Additional file 10: Figure S6 An alignment of select SET-domain containing histone methyltransferases with the protein translation of SET encoding gene fragments carried by some *Helentrons*. A protein alignment of the N- and C-terminal subregions of the SET domain (and SET-C, respectively) and the variable insert regions (SET-I) are shown. Identical residues are shaded in black and conservative changes are shaded in gray. Regions involved in binding to the cofactor product AdoHcy are indicated with green, and the three highly conserved sequence regions are indicated with a blue bar below the aligned sequences. The invariant tyrosine residue implicated to function as a general base for catalysis is indicated with a black star below the alignment. The insert region shows no structural conservation [50]. The various sequences used for alignment are histone H3 methyltransferase Clr4 from *Schizosaccharomyces pombe* (NP_595186.1), histone-lysine N-methyltransferase SUV39H1 isoform 2 from *Homo sapiens* (4507321:145-412), histone H3 methyltransferase DIM-5 from *Neurospora crassa*, (AAL35215.1), SET1 from *Oryza sativa* (AAK28975.1) putative histone-lysine N-methyltransferase from *Phytophthora infestans* (XP_002999311.1), and *Helentrons* from *P. cambivora* (AUVH01093707.1|: 299-12077), *P. capsici* (ADVJ01006715.1|:c14578-831), and *P. infestans* (see Additional file 8). Click here for file
