# Supplementary material for: DINE-1, the highest copy number repeats in Drosophila melanogaster are non-autonomous endonuclease-encoding rolling-circle transposable elements (Helentrons)
Source: Mob DNA. 2014 Jun 4;5:18. doi: 10.1186/1759-8753-5-18 (PMC4067079; doi:10.1186/1759-8753-5-18)
Supplement: Additional file 1: Table S1 — Distribution and copy number of DINE-1-like elements. [file 1759-8753-5-18-S1.pdf]

Additional file 1: Table S1. Distribution and copy number of *DINE-1*-like elements

**Name of the element**	**Organism**	**Copy number**	**Citation**
DINE-1	*D. willistoni*<br>*D. melanogaster* D. simulans, D. sechellia. D. grimshawi*<br>*D. yakuba, D. ananassae, D. mojavensis*<br>*D. erecta, D. persimilis, D. pseudoobscura*<br>*D. virilis*	6,297<br>334-502<br>*114 kbp<br>>5000<br>>1000<br>>3000	Yang and Barbash 2008<br>* Slawson *et al.* 2006 (dot chromosome)
SGM	*Drosophila subobscura, D. guanche*[§], and *D. madeirensis*	~ 10% of the genome[§]	[§]Miller *et al.* 2000
ISY	*D. miranda*[¶]<br>*D. pseudoobscura*[¶]	>1000[¶]	Steinmann and Steinmann 1993, Ellison and Bachtrog 2013[¶]
IS-amb, IS-gua, IS-sub	*D. ambigua, D. guanche, D. subobscura*	N/A	Hagemann *et al.* 1998
mini-me	*D. nigrodunni, D. dunni*	N/A	Wilder and Hollocher 2001
S812	*D. subobscura*	N/A	Marfany and Gonza`lez-Duarte 1992
GEM	*D. subobscura*	N/A	Vivas *et al.* 1999
PERI pSsp400	*D. buzzatti, D. koepferae, D. serido, D. borborema, D. seriema, D. antonietae und D. gouveai.*	N/A	Kuhn and Heslop-Harrison 2011
ISBu	*D. buzzatti,*	N/A	Cáceres *et al.*1999, Caceres *et al.* 2001, Negre *et al.* 2003
cDk27	*D. buzzatti, D. koepferae, D. serido*[#]*, D. borborema*[#]*, D. starmeri*[#]*, D. venezolana*[#]*, D. uniseta*[#]*, D. martensis*[#]	N/A	Marin *et al.* 1992, Marin and Fontdevilla 1996[#], Kuhn and Heslop-Harrison 2011
MINE-1	*Bombyx mori, Pectinophora gossypiella, Ostrinia nubilalis*[$]	~2700[$]	[$]Coates *et al.* 2010
MINE-2	*Bicyclus anynana, Helicoverpa armigera, Heliconius melpomene, H. numata, Spodoptera frugiperda*	N/A	Coates *et al.* 2011
NOVEL_NA	*Rhodnius prolixus*[¥]*, B. mori, Danaus plexippus*	881[¥]	[¥]Zhang *et al.* 2013
SGM	*Calliphora vicina*[€], *Lucilia cuprina*, *Musca domestica, Stomoxys calcitrans*	~5% (~30 kbp)[€]	Miller *et al.* 2000<br>[€]Negre and Simpson 2013

MgE	*Mytilus galloprovincialis*	N/A	Kourtidis *et al.* 2006
Pearl	*Crassostrea virginica, Anadara trapezia*	N/A	Gaffney *et al.* 2003
DTC84	*Donax trunculus*	1% (~89k)	Satovic and Plohl 2013
Tsp	*Strongylocentrotus purpuratus*	~1000	Cohen *et al.* 1985

Foot note: N/A denotes data not available. Related data in multiple columns are marked by same symbol.
