# Supplementary material for: DINE-1, the highest copy number repeats in Drosophila melanogaster are non-autonomous endonuclease-encoding rolling-circle transposable elements (Helentrons)
Source: Mob DNA. 2014 Jun 4;5:18. doi: 10.1186/1759-8753-5-18 (PMC4067079; doi:10.1186/1759-8753-5-18)
Supplement: Additional file 2: Figure S1 — Comparisons of the host flanking sequences of individual HINE insertions with paralogous sites in the genome that do not have the HINE insertion (empty sites). The first line is the host sequences with the Helentrons/HINE insertion. The second line is a paralogous site without the Helentron/HINE insertion. The black nucleotides represent the host sequence and underlined red nucleotides represent the transposable element. The accession and coordinates of the sequences are also given in black and the length of the transposable element is shown in red. (A-E) Empty sites of select Helentron/HINE insertions in Drosophila ananassae (HINE-Da-41A.2), D. willistoni (Helentron-Dw-41B.1), Culex quinquefasciatus (HINE-Cq-32A.1, HINE-Cq-32A.2), and Phytophthora infestans (proto-Helentron-Pi). [file 1759-8753-5-18-S2.pdf]

A

```
                  88538                   1    HINE-Da-41A.2.2 941                    89518
AAPP01017064.1:TTTGCAGAATTAAAGCAATT|TTTGAAGTGA...CACCCTTTTT|TTTTTTTTTTCTGTTGTACA
                  2473                                                           2512
AAPP01020495.1:TTTGCAGAATTAAAGCAATT--------------------------TTTCTTTTTTCTGTTGTACA
                  41576                                                          41615
AAPP01019905.1:TTTGCAGAATTAAAGCAATT--------------------------TTTCTTTTTTCTGTTGTACA
                  17994                                                          17955
AAPP01019784.1:TTTGCAGAATTAAAGCAATT--------------------------TTTCTTTTTTCTGTTGTACA
```

B

```
                                        Helentron-Dw-41B.1
                  19553                   1              11526                    31118
AAQB01008100.1:TGTCACTCATTTCAGTTTTT|TTTGAAGTGAA...CCCTCTTTT|TTTTTTTTACAGCAACTGTT
                  7920                                                            7760
AAQB01010620.1:TGTCACTCATTTCAGTTTTT--------------------------TTTTTTTTACAGCAACTGTT
                  745                                                             709
AAQB01007207.1:TGTCACTCATTTCAGTTTT------------------------------TTTTTTACAGCAACTGTT
```

C

```
                  14379                  1       HINE-Cq-32A.1                    16356
                                                          1937
AAWU01006445.1:AACCACTCATATTACCCATT|TTGACATAGG...TCTTGAACTT|TTTGGTAAAAAGTGAGGAAG
                  9881                                                            9721
AAWU01037158.1:AACCACTCATATTACCCATT--------------------------TTTGGTAAAAAGTGAGGAAG
                  6641                                                            6680
AAWU01047497.1:AACCACTCATATTACCCATT--------------------------TTTGGTAAAAAGTGAGGAAG
```

D

```
                  4519                    1      HINE-Cq-32A.2      1650                6207
AAWU01032153.1:GAAACCACTCTTTGCTGATTT|TTTGACATAT...TTGAACTTTTT|TTTTTTAAATTACCGTAAC
                  2957                                                              2918
AAWU01022713.1:AAAACCACTCTTTGCTGATTT---------------------------TTTTTTAAATTACCGTAAC
                  7392                                                              7353
AAWU01012390.1:AAAACCACTCTTTGCTGATTT---------------------------TTTTTTAAATTACCGTAAC
                  7949                                                              7911
AAWU01027758.1:AAAACCACTCTTTGCTGATTT---------------------------TTT-TTAAATTACCGTAAC
                  17561                                                             17523
AAWU01002241.1:AAAACCACTCTTTGCTGATTT---------------------------TTTTTTAA-TTACCGTAAC
```

E

```
                                        proto-Helentron-Pi
                  32071                  1               15065                      16967
AATU01002161.1:CAGGTTCAAAACAGTGTTTA|TTGGGGCAAA...TGCCATACTAG|TTTTAAAAAATACTACGTGCC
AATU01008688.1:CAGGTTCAAAACAGTGTTTA----------------------------TTTAAAAAAATACTACGTGCC
                  6917                                                              6878
```
