# Supplementary material for: DINE-1, the highest copy number repeats in Drosophila melanogaster are non-autonomous endonuclease-encoding rolling-circle transposable elements (Helentrons)
Source: Mob DNA. 2014 Jun 4;5:18. doi: 10.1186/1759-8753-5-18 (PMC4067079; doi:10.1186/1759-8753-5-18)
Supplement: Additional file 3: Figure S2 — Comparison of the host flanking sequences of multiple HINE insertions and insertion free sites (empty sites) in the genome. The underlined sequences in red represent the HINEs and black nucleotide represent the host sequence. The accession and coordinates of the sequences are also given in black. (A) Multiple HINE-Da-41A insertions with their flanking sequences in the Drosophila ananassae genome. (B) Empty sites for each HINE-Da-41A insertion. The first line is the host sequence with the HINE-Da-41A insertion. The second line is a paralogous site without the HINE insertion. (C) Multiple HINE-Mo-4C insertions with flanking sequences in the Metaseiulus occidentalis genome. (D). Empty sites for each HINE-Mo-4C insertion. The first line is the host sequence with the HINE-Mo-4C insertion. The second line is a paralogous site in the genome without the HINE insertion. [file 1759-8753-5-18-S3.pdf]

A

HINE-Da-41A

```
AAPP01017534.1:c8924-7855     AGCTTACACACTTTTT|GAAGTGAAAC---TGACACACCC|TTTTTTTTTCTCATAAGAAT
AAPP01017702.1:31670-32709    CTTTCCTTTCTTTTTT|GAAGTGAAAC---TGACACACCC|TTTTTTTTATTATACTATTA
AAPP01017064.1:88509-89548    AATTAAAGCAATTTTT|GAAGTGAAAC---TGACACACCC|TTTTTTTTTTTTTTTCTGTT
AAPP01018870.1:c2354-1315     TTTTAGTACATATATT|GAAGTGAAAC---TGACACACCC|TTTTTTTTTTTGAAATATAT
AAPP01016446.1:13137-14176    TCCAGGGGTTTTTTTT|GAAGTGAGAC---TGACACACCC|TTTTTTTTTAAATAAAACCG
```

B

HINE-Da-41A

```
AAPP01017534.1:c8924-7885     AGCTTACACACTTTTT|GAAGTGAAAC---TGACACACCC|TTTTTTTTTCTCATAAGAATG
AAPP01019994.1:7142-7240      AGCTTACACACTT----------------------------------TCTTCTCATAAGAATG

AAPP01017702.1:31670-32709    CTTTCCTTTCTTTTTT|GAAGTGAAAC---TGACACACCC|TTTTTTTTATTATACTATTA
AAPP01019829.1:48873-48974    CTTTCCTTTCTTTT-------------------------------TTTTTATTATACTATTA

AAPP01017064.1:88509-89546    AATTAAAGCAATTTTT|GAAGTGAAAC---TGACACACCC|TTTTTTTTTTTTTTTCTGTTG
AAPP01020495.1:2444-2540      AATTAAAGCAATT----------------------------------TTTCTTTTTTCTGTTG

AAPP01018870.1:c2354-1315     TTTTAGTACATATATT|GAAGTGAAAC---TGACACACCC|TTTTTTTTTTTGAAATATATG
AAPP01018626.1:c46436-46340   TTCTAGTACACATAA--------------------------------TTTTTGAAATATATG

AAPP01016446.1:13137-14176    TCCAGGGGTTTTTTTT|GAAGTGAGAC---TGACACACCC|TTTTTTTTTAAATAAAACCG
AAPP01016446.1:5512-5605      TCCAGGGGTTTTT--------------------------------TTTTTTCAATAAAACCG
```

C

HINE-Mo-4C

```
AFFJ01003087.1:7700-8536      CAGCTTGATAGTTTT|GACGTGAAAC---TACCTCTGAA|TTTTTTAAATTCAAGAGA
AFFJ01002227.1:497216-498068  GTGAAACGTCTTTTT|GACGTGAAAC---TACCTCTGAA|TTTTTTATTATATCCACC
AFFJ01003063.1:c5422-4551     ATCTCAAGTTTTTTT|GACGTGAAAC---TACCTCTGAA|TTATTTTTAAATTTTGA
```

D

HINE-Mo-4C

```
AFFJ01003087.1:7700-8536      TCAGCTTGATAGTTTT|GACGTGAAA----TACCTCTGAA|TTTTTTAAATTCAAGAGA
AFFJ01003317.1:c41365-41265   TCAGCTTGACGGT------------------------------TTTCAAATTTAAGAGT

AFFJ01002227.1:497216-498068  AATCTGGATTCTTTTT|GACGTGAAAC---TACCTCTGAA|TTTTTTATTATATCCACC
AFFJ01002228.1:3579-3678      AATCTGGATTCTT-------------------------------TTCATTATATCCACC

AFFJ01003063.1:c5422-4551     TATCTCAAGTTTTTTT|GACGTGAAAC---TACCTCTGAA|TTATTTTTTAAA-TTTTGAA
AFFJ01000047.1:c817-713       TATTTCAAGTTTT----------------------------------TTCAAATTTTTGAA
```
