# Supplementary material for: DINE-1, the highest copy number repeats in Drosophila melanogaster are non-autonomous endonuclease-encoding rolling-circle transposable elements (Helentrons)
Source: Mob DNA. 2014 Jun 4;5:18. doi: 10.1186/1759-8753-5-18 (PMC4067079; doi:10.1186/1759-8753-5-18)
Supplement: Additional file 4: Figure S3 — The protein alignment of the Rep motif of representative Helentrons and Helitrons and a phylogenetic tree based on an alignment of the most conserved Rep motifs/Helicase domains. (A) An alignment of the Rep motif of Helentrons from 12 species, Helitrons from seven species and representative plasmids and viruses that utilizes rolling-circle replication (RCR). Black asterisks above the alignment denote the positions of the two histidines and two tyrosines known to be critical for catalytic activity of the RC elements. Identical residues are shaded in black and conservative changes are shaded in gray. Amino acids that distinguish Helentrons from Helitrons are boxed in red. The accession and coordinates of the different sequences used in the alignment are: Helentrons from Metaseiulus occidentalis Mite-1 (AFFJ01001714.1:c5449-8790), Mite-3 (AFFJ01002321.1:c999-4343) Culex quinquefasciatus (AAWU01024641.1:12176- 15496), platyfish Xiphophorus maculatus (ABB05534.1), fungi Mucor circinelloides (EPB86818.1), acornworm Saccoglossus kowalevskii (XP_002741052.1), Phytophthora infestans (AATU01002056.1:10099-12180), Nematostella vectensis (Helitron-1_NV) [30], sea urchin Strongylocentrotus purpuratus (AAGJ04076666.1:8326-11865), Danio rerio (DAA01284.1), Frog Xenopus tropicalis (AAMC02019010.1: 25350- 33598) Drosophila willistoni (AAQB01006357.1:146323-152490), D. ananassae (AAPP01019845.1:107830-112664), D. yakuba (AAEU02001960.1:c3447-10117). Helitrons from mite M. occidentalis (AFFJ01001759.1:1748-4869), D. ananassae (AAPP01018364.1:33765-39124), Aphid Acyrthosiphon pisum (AC202211.4:97955-103017), Myotis lucifugus (AAPE02018439.1:1503-5146), Bombyx mori Helianu_Bm1[54], Oryza sativa japonica (AAM92800.1), and Arabidopsis thaliana (AtHEL2p) [2]. SVTS, Spiroplasma plectro virus (AAF18311.2); Rep_SC, Streptomyces cyaneus plasmid (BAA34784.1); Rep_BB, Bacillus borstelensis plasmid (BAA07788.1); Rep_AA, Actinobacillus actinomycetemcomitans plasmid (AAC37125.1); Pf3, Pseudomonas a [file 1759-8753-5-18-S4.pdf]

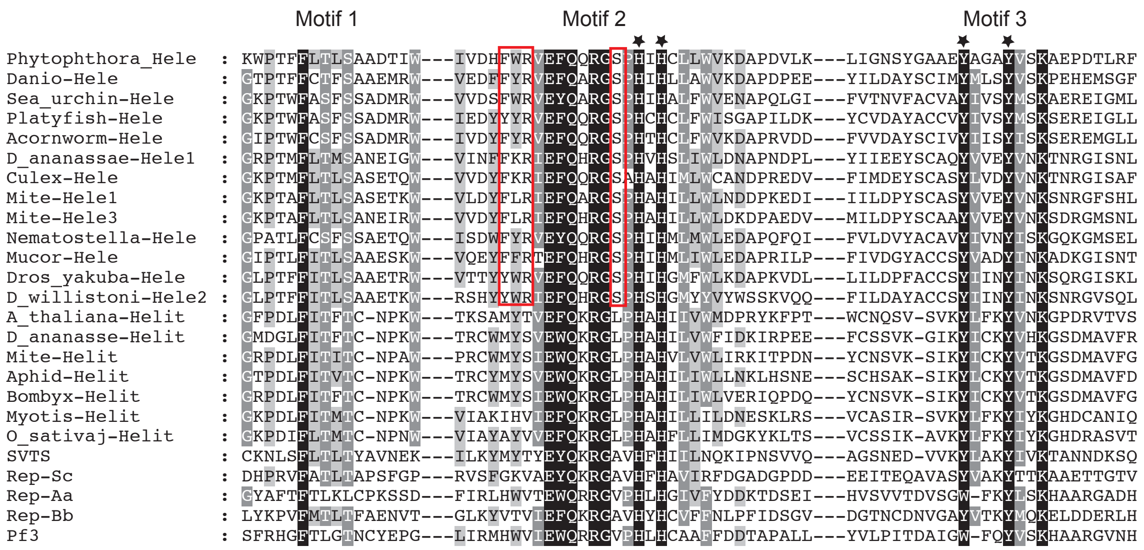

Motif 1
Motif 2
Motif 3
Phytophthora_Hele : KWPTFFLTLSAADTIW---IVDHFWRVEFQQRGSPHIHCLLWVKDAPDVLK---LIGNSYGAAEYAGAYVSKAEPDTLRF
Danio-Hele : GTPTFFCTFSAAEMRW---VEDFFYRVEFQARGSPHIHLLAWVKDAPDPEE---YILDAYSCIMYMLSYVSKPEHEMSGF
Sea_urchin-Hele : GKPTWFASFSSADMRW---VVDSFWRVEYQARGSPHIHALFWVENAPQLGI---FVTNVFACVAYIVSYMSKAEREIGML
Platyfish-Hele : GKPTWFASFSSADMRW---IEDYYYRVEFQQRGSPHCHCLFWISGAPILDK---YCVDAYACCVYIVSYMSKSEREIGLL
Acornworm-Hele : GIPTWFCSFSSADMRW---IVDYFYRVEFQQRGSPHTHCLFWVKDAPRVDD---FVVDAYSCIVYIISYISKSEREMGLL
D_ananassae-Hele1 : GRPTMFLTMSANEIGW---VINFFKRIEFQHRGSPHVHSLIWLDNAPNDPL---YIIEEYSCAQYVVEYVNKTNRGISNL
Culex-Hele : GKPTMFLTLSASETQW---VVDYFKRIEFQQRGSAHAHIMLWCANDPREDV---FIMDEYSCASYLVDYVNKTNRGISAF
Mite-Hele1 : GKPTAFLTLSASETKW---VLDYFLRIEFQARGSPHAHILLWLNDDPKEDI---IILDPYSCASYVVEYVNKSNRGFSHL
Mite-Hele3 : GKPTAFLTLSANEIRW---VVDYFLRIEFQHRGSPHAHILLWLDKDPAEDV---MILDPYSCAAYVVEYVNKSDRGMSNL
Nematostella-Hele : GPATLFCSFSSAETQW---ISDWFYRVEYQQRGSPHIHMLMWLEDAPQFQI---FVLDVYACAVYIVNYISKGQKGMSEL
Mucor-Hele : GIPTLFITLSAAESKW---VQEYFFRTEFQHRGSPHIHMLIWLEDAPRILP---FIVDGYACCSYVADYINKADKGISNT
Dros_yakuba-Hele : GLPTFFITLSAAETRW---VTTYYWRVEFQQRGSPHIHGMFWLKDAPKVDL---LILDPFACCSYIINYINKSQRGISKL
D_willistoni-Hele2 : GLPTFFITLSAAETKW---RSHYYWRIEFQHRGSPHSHGMYYVYWSSKVQQ---FILDAYACCSYIINYINKSNRGVSQL
A_thaliana-Helit : GFPDLFITFTC-NPKW---TKSAMYTVEFQKRGLPHAHIIVWMDPRYKFPT---WCNQSV-SVKYLFKYVNKGPDRVTVS
D_ananasse-Helit : GMDGLFITFTC-NPKW---TRCWMYSVEWQKRGLPHAHILVWFIDKIRPEE---FCSSVK-GIKYICKYVHKGSDMAVFR
Mite-Helit : GRPDLFITITC-NPAW---PRCWMYSIEWQKRGLPHAHVLVWLIRKITPDN---YCNSVK-SIKYICKYVTKGSDMAVFG
Aphid-Helit : GTPDLFITVTC-NPKW---TRCYMYSVEWQKRGLPHAHILIWLLNKLHSNE---SCHSAK-SIKYLCKYVTKGSDMAVFD
Bombyx-Helit : GRPDLFITFTC-NPKW---TRCWMYSIEWQKRGLPHAHILIWLVERIQPDQ---YCNSVK-SIKYICKYVTKGSDMAVFG
Myotis-Helit : GKPDLFITMTC-NPKW---VIAKIHVIEFQKRGLPHAHILLILDNESKLRS---VCASIR-SVKYLFKYIYKGHDCANIQ
O_sativaj-Helit : GKPDIFLTMTC-NPNW---VIAYAYVVEFQKRGLPHAHFLLIMDGKYKLTS---VCSSIK-AVKYLFKYIYKGHDRASVT
SVTS : CKNLSFLTLTYAVNEK---ILKYMYTYEYQKRGAVHFHIILNQKIPNSVVQ---AGSNED-VVKYLAKYIVKTANNDKSQ
Rep-Sc : DHPRVFATLTAPSFGP---RVSFGKVAEYQKRGAVHFHAVIRFDGADGPDD---EEITEQAVASYVAKYTTKAAETTGTV
Rep-Aa : GYAFTFTLKLCPKSSD---FIRLHWVTEWQRRGVPHLHGIVFYDDKTDSEI---HVSVVTDVSGW-FKYLSKHAARGADH
Rep-Bb : LYKPVFMTLTFAENVT---GLKYVTVIEFQKRGAVHYHCVFFNLPFIDSGV---DGTNCDNVGAYVTKYMQKELDDERLH
Pf3 : SFRHGFTLGTNCYEPG---LIRMHWVIEWQRRGVPHLHCAAFFDDTAPALL---YVLPITDAIGW-FQYVSKHAARGVNH

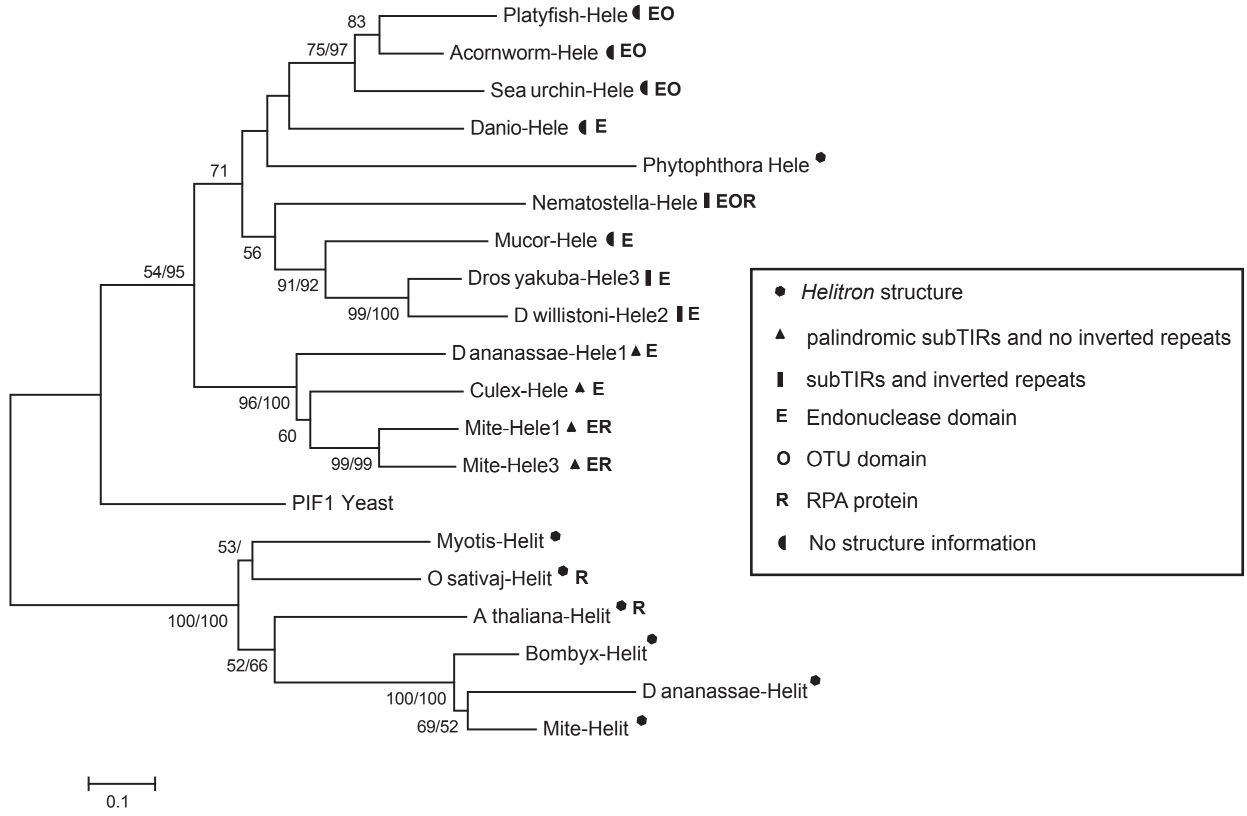

83
75/97
71
54/95
56
91/92
99/100
96/100
60
99/99
53/
100/100
52/66
100/100
69/52
Platyfish-Hele EO
Acornworm-Hele EO
Sea urchin-Hele EO
Danio-Hele E
Phytophthora Hele
Nematostella-Hele EOR
Mucor-Hele E
Dros yakuba-Hele3 E
D willistoni-Hele2 E
D ananassae-Hele1 E
Culex-Hele E
Mite-Hele1 ER
Mite-Hele3 ER
PIF1 Yeast
Myotis-Helit
O sativaj-Helit R
A thaliana-Helit R
Bombyx-Helit
D ananassae-Helit
Mite-Helit
0.1
Helitron structure
palindromic subTIRs and no inverted repeats
subTIRs and inverted repeats
E Endonuclease domain
O OTU domain
R RPA protein
No structure information
