# Supplementary material for: DINE-1, the highest copy number repeats in Drosophila melanogaster are non-autonomous endonuclease-encoding rolling-circle transposable elements (Helentrons)
Source: Mob DNA. 2014 Jun 4;5:18. doi: 10.1186/1759-8753-5-18 (PMC4067079; doi:10.1186/1759-8753-5-18)
Supplement: Additional file 5: Figure S4 — Protein alignment of the PIF1 helicase from Helentrons, Helitrons, and select organisms. The eight conserved motifs of the PIF1 family of helicases from Helentrons, Helitrons, yeast (P07271), baculovirus (Q9YMS4), TRAA_RHISN, Rhizobium sp (P55418.1), (P55418.1), and T4 phage (P32270). The accession and coordinates of the Helentrons and Helitrons used in the alignment are: Helentrons from Metaseiulus occidentalis Mite-1 (AFFJ01001714.1:c5449-8790), Mite-3 (AFFJ01002321.1:c999-4343) Culex quinquefasciatus (AAWU01024641.1:12176-15496), platyfish Xiphophorus maculatus (ABB05534.1), fungi Mucor circinelloides (EPB86818.1), acornworm Saccoglossus kowalevskii (XP_002741052.1), Phytophthora infestans (AATU01002161.1: 20532-18847), Nematostella vectensis (Helitron-1_NV) [30], sea urchin Strongylocentrotus purpuratus (AAGJ04076666.1:8326-11865), Danio rerio (DAA01284.1), Frog Xenopus tropicalis (AAMC02019010.1: 25350- 33598), Drosophila willistoni (AAQB01006357.1:146323-152490), D. ananassae (AAPP01019845.1:107830-112664), D. yakuba (AAEU02001960.1:c3447-10117). Helitrons from Mite M. occidentalis (AFFJ01001759.1:1748-4869), D. ananassae (AAPP01018364.1:33765-39124), Aphid Acyrthosiphon pisum (AC202211.4:97955-103017), Rhodnius prolixus HeligloriaAi_Rp1 (ACPB01050589.1:10663-14351), Bombyx mori Helianu_Bm1[54], Oryza sativa japonica (AAM92800.1), and Arabidopsis thaliana (AtHEL2p) [2]. [file 1759-8753-5-18-S5.pdf]

```
                                  I             Ia           II             III
Mite-Hele1              : LTGPAGCGKTYTLKAL---YISTATTGKAATAL---CIIIDEISMCSSHVF---GLDLFACGDLKQLPPV
Mite-Hele3              : LTGPAGCGKTFTLKAI---YIATATTGKAAVGL---CVIIDEISMCSSHLL---GLDLIACGDLRQLPPV
D_ananasse-Hele1        : LTGPAGCGKTFTIKLL---YIACASTGKAAVAT---VIVIDEVSMIGAEML---GMNMIFIGDLRQLPPV
Culex-Hele              : LTGPAGSGKTFTLRLM---YIACASTGKAAVNI---LHIVDEVSMLSAGNF---DQDVT--GDLHQLNPV
Phytophthora_Hele       : INGEGGSGKSWLIRHI---VLLLAHQGTAAFNI---LVIIDEFSMISCGML---GRDVYFTGDSAQLDPV
Frog-Hele               : VSGGAGCGKSHVIKCI---VLLTAFTGTAVFNI---ILIIDEVSRISKDLF---GISCLAVGDFFQLRVE
Danio_Hele1             : VSGGAGCGKSHVIKCV---VILSAFTGTAAFNI---ILIIDEISMISKDFF---GISVIVVGDFYQLPPP
Sea_urchin-Hele         : LTGGAGTGKSHLIKCL---VLKMAPTGVAAYNI---ILIIDEVSMVNQKLM---GVSVIAVGDMYQLPPV
Platyfish-Hele          : ITGGAGTGKSHLIKAI---VLLTAPTGIAAYNL---LLIIDEISMVDHNLL---NISIIVVGDMYQLPPV
Acornworm-Hele          : LCGGAGTGKSHLIKAL---VLLTAPTGVAAFNI---ILIIDEISMVDKKLL---GVSVVAVGDFYQLPPV
Nematostella-Hele       : LSGGAGVGKSHVTKAL---ILMLAPTGKAAYNI---LIFVDEISMVGNTMF---GVSIVAIGDLFQLQPV
Mucor-Hele              : VTGGAGTGKSMLINTL---VLLCAPTGIAAFNI---VVIIDEISMVGSLQF---GISIFVFGDFLQLPPV
D_yakuba-Hele3          : VGGGAGVGKSRLISTI---ILLCAPTGKAAFGI---LIIIDEISMVGATMF---GISVIVFGDLRQLRPV
D_willistoni-Hele2      : IGGGAGVGKSRLISTI---VLLCAPTGKAAFGI---LIIIDEISMVGAKML---GISIIVFGDLKQLSPV
Bombyx-Helit            : LDAPGGTGKTFLMSLV---AVAVASSGIAATLL---IIIWDECTMAHKRAL---GAMILLSGDFRQILPV
Mite-Helit              : LDAPGGTGKTFLISLI---GLAMASSGIAATLL---LIIWDECTMAHKRAL---GALILLSGDFRQTLPV
Rhodnius-Helit          : LDAPGGTGKTFLISLI---ALAVASSGIAATLL---IIIWDECTMAHKYSL---GAILLLSGDFRQTLPV
Myotis-Helit            : LDGPGGSGKTYLYKVL---VLPTASTGIAANLL---LLIIDECTMASSHAI---GKVLLLGGDFRQCLSI
Dr_ananassae-Helit      : LDAPGGTGKTFVISLI---ALAVASSGIAATLL---LIIWDECTMAHCTMT---GTLLVLSGDFRQTLPV
O_sativaja-Helit        : VDGPGGTGKTFLYKAL---AVATATSGVAASIM---LIIWDEASMTKRQAV---GKTVVFGGDFRQVLPV
A_thaliana-Helit        : LYGFGGTWKTFLWKVL---CLNVASSGIASLLL---LIIWDEAPMMSKYCF---GKVILFGGDFRQILPV
TRAA_RHISN              : VIGRAGAGKTTMMKAA---VVGGALAGKAAEGL---IFVLDEAGMVSSRQM---GAKLVLVGDPEQLQPI
Hel_T4                  : INGPAGTGKTTLTKFI---IILAAPTHAAKKIL---VLICDEVSMYDRKLF---WCTIIGIGDNKQIRPV
PIF1_Yeast              : YTGSAGTGKSILLREM---VAVTASTGLAACNI---ALVVDEISMLDAELL---GIQLIFCGDFFQLPPV
Baculovirus             : VSGSAGTGKSALLMAL---VLVAAYTNLAARNV---CVIIDEISMIPAKML---GVNVIVFGDLYQLPPV

                           IV        IV/V                  V                 VI
Mite-Hele1              : ---LVQVVR---GYPYMITVNVDVEDGLVNG---VPWTIHKSQGGTFNR-IV---YVALSRVTSLEGLYI
Mite-Hele3              : ---LTRVVR---NYPYIITANVDVEDGLVNG---CALTIHKSQGGTFDR-IV---YVAMSRVTSVDGLFL
D_ananasse-Hele1        : ---LTEVMR---NKPYLVTTNIDVTDGLANG---CAMTIHKSQGGTYDS-VV---YVALSRVTSPQGLYV
Culex-Hele              : ---LDQVMR---GKPYMITTNIDGDDNLVNG---CSMTIHKSQGGTFSK-VV---YVALSRVTSLEGLYL
Phytophthora_Hele       : ---LTSQNR---GAPITLTYNIAQAAGLCNG---YAMTVHKVQGLTCSC-VV---YVALSRITHRNLIVI
Frog-Hele               : ---LTELMR---GVCVMVTRNLDVEDGIVNG---YACTAHKVQGMTMQSAVV---YVALSRTTSLSGLHI
Danio_Hele1             : ---LTEIMR---GVRIMMIRNLDVEDGLVNG---YACTAHKVQGMTMQSAVV---YVALSRTTSLGGLYI
Sea_urchin-Hele         : ---LEDIMR---NARVMILKNIDVSKGLTNG---YACTIHKVQGSTLDKAVV---YVGLSRVTSLSGLVI
Platyfish-Hele          : ---LTEIVR---GARVMICKNVDVGDGLVNG---WACTVHKVQGLTVDEAVV---YVAISRVRSVLGLTI
Acornworm-Hele          : ---LSDIVR---GARVMITKNIDVSDGLVNG---WACTVHKVQGLTVEEAVV---YVALSRVKSLSGLVI
Nematostella-Hele       : ---LKEIMR---GERTEISLNTRNDDGMTNG---AAKTIHRSQGDTESRIVV---YVGLSRVTTIDGLYI
Mucor-Hele              : ---LTQIMR---GGKYIISRNVKTSDGIVNG---ESLTVHKSQGQTYGSVVV---YVACSRATASSGLRI
D_yakuba-Hele3          : ---LTEIMR---TAKYMMTVNVDTSDGLVNG---EAITIHKSQGATYSKVVV---YVACSRATTAAGLFI
D_willistoni-Hele2      : ---LTEIMR---QAKYMVTVNVDTSDGLVNG---EAITIHKSQGATYSKVAV---YVGCSRATSASGLFL
Bombyx-Helit            : ---LTTNMR---GSVVIMLRNINQPK-LCNG---FAMTINKSQGQSLKVCGL---YVACSRVGRPSALFV
Mite-Helit              : ---LRINMR---GVPIILLRNINPPR-ICNG---FAMTINKSQGQTLEVCGL---YVACSRVGNPSNLYV
Rhodnius-Helit          : ---LTINMR---GSPIILLRNLNPPQ-LCNG---FAMSINKSQGQTMSICGL---YVACSRVGKPSNLFV
Myotis-Helit            : ---LTTNMR---GAIIMILRNLNSKWGLCNG---FAMTINKSQGQTLDRVGI---YVAFSRVRRACDVKV
Dr_ananassae-Helit      : ---QKINMR---GSPIILLRNLNPPR-LCNG---FAMTINKSQGQTMSVCWI---TLFFTRTIIRGILSS
O_sativaja-Helit        : ---LVTNMR---NCPVILLRNIDPANGLCNG---FAMTINKAQGQTIPNVGV---YVALSRATARMNIKI
A_thaliana-Helit        : ---LTKNMR---GCPVMLLRNLDPHGGLMNG---FAMTINKSQGQSLGNVGI---YVAMSRVKSKAGLKV
TRAA_RHISN              : ---LETIYR---GDQIVFLKN-EGALGVKNG---YATTIHKSQGATVDRVKV---YVAMTRHREDLGVYY
Hel_T4                  : ---LTEVKR---GQLVRIIEAEYTSTFVKAR---PASTFHKAQGMSVDRAFI---YVGVTRGRYD-VFYV
PIF1_Yeast              : ---LQKVFR---GAQVMMVKNLDAT--LVNG---WSLSIHKSQGQTLPKVKV---YVALSRAVSREGLQV
Baculovirus             : ---LTENMR---GARVMITHTTAE---FCNG---WAVTIHKAQGMTLKNLIV---YVALSRTVHSSGLKL
```
