# Supplementary material for: DINE-1, the highest copy number repeats in Drosophila melanogaster are non-autonomous endonuclease-encoding rolling-circle transposable elements (Helentrons)
Source: Mob DNA. 2014 Jun 4;5:18. doi: 10.1186/1759-8753-5-18 (PMC4067079; doi:10.1186/1759-8753-5-18)
Supplement: Additional file 6: Figure S5 — An alignment of the apurinic/apyrimidinic endonuclease alignment encoded by Helentrons, non-LTR retrotransposons and select cellular proteins. A protein alignment of the endonuclease domains of Helentrons from 12 species, non-LTR retrotransposons from five species, and three cellular endonucleases. The accession and coordinates of the different sequences used in the alignment are: Helentrons from Metaseiulus occidentalis Mite-1 (AFFJ01001714.1:c5449-8790), Mite-2 (AFFJ01002369.1:4460-5251) Culex quinquefasciatus (AAWU01024641.1:12176- 15496), platyfish Xiphophorus maculatus (ABB05534.1), fungi Mucor circinelloides (EPB86818.1), acornworm Saccoglossus kowalevskii (XP_002741052.1), sea urchin Strongylocentrotus purpuratus (AAGJ04076666.1:8326-11865), Danio rerio (DAA01284.1), Frog Xenopus tropicalis (AAMC02019010.1: 25350-33598), Drosophila willistoni (AAQB01006357.1:146323-152490), D. ananassae (AAPP01019845.1:107830-112664), D. yakuba (AAEU02001960.1:c3447-10117). The cellular endonucleases are from Bos taurus APEX1_BOVIN (P23196.2), Homo sapiens APEX (AAB26054.1), Escherichia coli APEX3 (AAC74819.1). The non-LTR are from Daphnia pulex (EFX61861.1), Trypanosoma cruzi (CAB41692.1), Danio rerio (BAE46430.1), Oryzias latipes ReO_6 (BAB83841.1), Nematostella vectensis -Rex1_CR1, and C.elegans-Frodo_CR1 [38]. [file 1759-8753-5-18-S6.pdf]

```
                              I              II ★           III             IV
Mite-Hele1        : WTIVNVNVQSL---ADLLVTTETWMH---RSGGVAIYRK---EFILAAVYMHQGN
Mite-Hele2        : IIILNFNVQSL---SHLLCLSETWMK---RTGGVAIYAN---PILLVVVYISPGT
Danio_Hele1       : LIVVHHNTEGL---CDIFCVTETHLS---DCGGVAIYCK---TATIAAVYRPPDY
Mucor-Hele        : ILVLSHNIQSL---ASVLLFSETWTV---SATGACCYVN---SKLFASVYMSPNA
Bombyx-Hele       : IIVFSLNCQSL---SHVLMLSETWLS---RGGGVCIYHN---EIIMVTVYISVNT
D_ananassae-Hele  : MTIFSFNCQSI---SDILMLSETWLD---RAGGVQHVVV---TIIMVSIYISPGK
D_yakuba-Hele3    : LHIFFHNTESL---SSLLCFVEASTY---PKRGILVFIR---SLGILVLYKSPTY
Culex-Hele2       : CTLMTINVQSL---VDYLALSETWLD---RSGGVAIYRK---EVLLFSVYIIPGT
Nematostella-CR1  : PNVIVSNVMSL---ADLVFITETWLK---MHGGVCAYIK---CIIAAAIYHPPSA
Frog-Hele         : LTILHHNTEGL---SDILCLMETHLS---DGGGVAIFCR---NATIAAVYRPPQY
Platyfish-Hele    : FTVFLMNVQSL---PKCIAITETWVS---QHGGVGIYCA---NMVLGVIYRPPLY
Acornworm-Hele1   : YTITLHNIEGI---SDFICLTETWLT---AHGGVGVYIS---HFTVAIIYRPQSY
Sea_urchin-Hele   : FSLVMQNVQGL---VDCICLTETWIN---SHGGVAMYIK---SVVISVVYRPPSY
Oryzias-CR1       : PAIHLGNVRSL---SSALCFTETWLS---KGGGTCFYIN---SFVLVGVYIPPQA
Danio-CR1_2       : LSVGLWNCQSA---YNLMALTETWLR---RGGGTGLLIS---YINVVVIYRPPGK
C_elegans-CR1     : LSFLASNVRSS---YSVICLQETWLT---RGGGVCTVVN---PLRVINVYRPPTC
Daphnia-L2        : AKLCLLNARSI---PDILAITETWLT---RGGGVAVIFK---AIRLLVVYRPPAS
Trypanosoma-L1    : IAVLQMNVSCL---ADIIAIQETWKS---KGGGVAVLVR---DLIVASAYMRPPP
Exo-Ecoli         : MKFVSFNINGL---PDVIGLQETKVH---GHYGVALLTK---NVTVINGYFPQGE
APEX1_Bovine      : LKICSWNVDGL---PDILCLQETKCS---GYSGVGLLSR---AFVLVTAYVPNAG
APEX-Human        : LKICSWNVDGL---PDILCLQETKCS---GYSGVGLLSR---SFVLVTAYVPNAG

                                  V                 VI ★            VII ★
Mite-Hele1        : ---PMLVIGDFNTSNDNRK---TTLGGTCIDLTFAR---SYFSYHRPVFNKIQY
Mite-Hele2        : ---STILCGDFNWNIQDQT---TTRDRSCIDLIFSN---SYFSYHKPVFFGVKD
Danio_Hele1       : ---PIIVCGDFNEDHLSSG---TTEKQTLLDHIYVS---TYYSYHNPVYCVLPK
Mucor-Hele        : ---SLVIGGDFNVDFGPNA---TSKNGTFIDNIFTN---SFTSLHEPLYIEIPN
Bombyx-Hele       : ---PLILGGDFNIDFNKED---TTRYDTTIDAMFSR---SYFSYHKPIVSVFND
D_ananassae-Hele  : ---PMIISGDFNVNFAREE---TTRHGTTIDAVFAR---SHFSYHNPIITTLGN
D_yakuba-Hele3    : ---NCLVLGDFNLCLHSIS---TTNCNTHIDWAFSN---TTYSYHSGILVSVRE
Culex-Hele2       : ---PMVVTGDFNVDVSKRE---TTNRGTVLDLTFTR---CYFSYHRPMLSVLRT
Nematostella-CR1  : ---GILLTGDFNRLDVSGL---TR-KDATLDLILTN---FGLSDHNTVLAAAID
Frog-Hele         : ---PVIICGDFNEDLLSPG---TTEKDTLLDQIYVS---TYHSYHNPVYSILRK
Platyfish-Hele    : ---TIALIGDFNDNILKSS---TTEKDTLIDHVYVK---TYFSDHEGIMCGFSL
Acornworm-Hele1   : ---GSIVLGDFNENVLVCS---TTDSGTLIDHAYVR---TYFGSHNAVCLKV--
Sea_urchin-Hele   : ---RCIVMGDFNEDLFKGS---TTEKGTLIDHVYSK---TYYSYHEAVKIAF--
Oryzias-CR1       : ---LVITLGDFNRANLKAE---TR-GLNILDHCYTT---LGLSDHCLVYLIPTY
Danio-CR1_2       : ---PLLVLGDFNIYVDKPQ---THKSGNQLDLIYTR---LQISDHFLLSLNIHI
C_elegans-CR1     : ---NSIIVGDFNAGDINWS---PTRKTKVLDLVLSN---IGSSDHNRIEFSLDL
Daphnia-L2        : ---KLLIVGDFNLSIRDQP---THEGGSILDLVFTR---GFFSDHRPVLVSLSC
Trypanosoma-L1    : ---PLLLCGDFNMHHPQWE---GTRERSCIDLTWSK---SPLSDHYVLTFTLHQ
Exo-Ecoli         : ---PVLIMGDMNISPTDLD---DDNRGLRIDLLLAS---EKPSDHAPVWATFRR
APEX1_Bovine      : ---PLVLCGDLNVAHEEID---SKNVGWRLDYFLLS---ALGSDHCPITLYLAL
APEX-Human        : ---PLVLCGDLNVAHEEID---SKNVGWRLDYFLLS---ALGSDHCPITLYLAL
```
