# Supplementary material for: DINE-1, the highest copy number repeats in Drosophila melanogaster are non-autonomous endonuclease-encoding rolling-circle transposable elements (Helentrons)
Source: Mob DNA. 2014 Jun 4;5:18. doi: 10.1186/1759-8753-5-18 (PMC4067079; doi:10.1186/1759-8753-5-18)
Supplement: Additional file 7: Table S2 — The subterminal inverted repeats (subTIRs) of Helentron-HINE families identified from different species. [file 1759-8753-5-18-S7.pdf]

Additional file 7: Table S2. The subterminal inverted repeats (subTIRs) of *Helentron-HINE* familes identified from different species.

Organism	Name of the element	subTIRs
Metaseiulus occidentalis	*Helentron-Mo-1*	GACGTGAAAACGT
	HINE-Mo-2	GCGTAGAACTACG
	HINE-Mo-3	ACGTGGGACACG
	HINE-Mo-4	GACGTGAAACGTCT
	HINE-Mo-5	CGTAGGACTGCGT
	HINE-Mo-6	ACGTGAACCACGT
	HINE-Mo-7	CGTCGAAGACGTCA
Culex quinquefasciatus	*Helentron-Cq-31*	TGACGAAGAACTTCG
	Helentron-Cq-32	GACATAGGACTA
	HINE-Cq-33	GCCTTCCTCACGTT
Drosophila ananassae	*Helentron-Da-41*	GAAGTGAAACTTCT
	*Helentron-Da-40**	ATACCCTTGCAG
D. willistoni	*Helentron-Dw-40**	ATACCCTTGCAA
	Helentron-Dw-41	GAAGTGAAAACTTC
D. yakuba	*Helentron-Dy-60.1**	ATACCCGTTACTC

* *DINE-1* elements corresponding to these *Helentrons* are described in Yang and Barbash 2008
