# Supplementary material for: DINE-1, the highest copy number repeats in Drosophila melanogaster are non-autonomous endonuclease-encoding rolling-circle transposable elements (Helentrons)
Source: Mob DNA. 2014 Jun 4;5:18. doi: 10.1186/1759-8753-5-18 (PMC4067079; doi:10.1186/1759-8753-5-18)
Supplement: Additional file 9: Table S3 — The presence of genes in Helentron families identified in this study or previously described in the literature. [file 1759-8753-5-18-S9.pdf]

Additional file 9 : Table S3. The presence of genes in *Helentron* families identified in this study or previously described in the literature.

	Organism	Zn finger	Rep	Helicase	Endonuclease	OTU	SET Domain	Histone 2A *	RPA	Transmembrane protein *
1	*Danio rerio* (Zebrafish), *Sphoeroides nephelus* (Pufferfish) *Ciona intestinalis* *C. savignyi*, *Gasterosteus aculeatus* (stickleback fish), *Strongylocentrotus purpuratus* (Sea urchin),	+	+	+	+	-	-	-	-	-
2	*Xiphophorus maculatus*, (Platyfish) *Oryzias latipes* (Medaka), *S. purpuratus, Branchiostoma floridae* (lancelet) , *D. rerio*	+	+	+	+	+	-	-	-	-
3	*Phytophthora infestans*	+	+	+	-	-	+	-	-	+
4	*Metaseiulus occidentalis*	+	+	+	+	-	-	-	+	-
5	*Nematostella vectens*	+	+	+	+	+	-	-	+	-
6	*Culex quinquefasciatus*	-	+	+	+	-	-	+	-	-
7	*Drosophila ananassae, D. willistoni*	+	+	+	+	-	-	-	-	-
8	*Xenopus tropicalis* (Frog)	+	+	+	+	+	-	-	-	-
9	*Chionodraco hamatus* (Ice fish), Nototheniid fishes, Bathydraconids fishes	+	+	+	+	+	-	-	-	-

* Denotes a possible gene capture, only found in one *Helentron* family. 1& 2 from Poulter *et al*. 2003, Zhou *et al*. 2006, 5&8 Kapitonov and Jurka 2007, 9. Cocca *et al*. 2011. 3,4,5,6 This study.
