# Supplementary material for: DINE-1, the highest copy number repeats in Drosophila melanogaster are non-autonomous endonuclease-encoding rolling-circle transposable elements (Helentrons)
Source: Mob DNA. 2014 Jun 4;5:18. doi: 10.1186/1759-8753-5-18 (PMC4067079; doi:10.1186/1759-8753-5-18)
Supplement: Additional file 10: Figure S6 — An alignment of select SET-domain containing histone methyltransferases with the protein translation of SET encoding gene fragments carried by some Helentrons. A protein alignment of the N- and C-terminal subregions of the SET domain (and SET-C, respectively) and the variable insert regions (SET-I) are shown. Identical residues are shaded in black and conservative changes are shaded in gray. Regions involved in binding to the cofactor product AdoHcy are indicated with green, and the three highly conserved sequence regions are indicated with a blue bar below the aligned sequences. The invariant tyrosine residue implicated to function as a general base for catalysis is indicated with a black star below the alignment. The insert region shows no structural conservation [50]. The various sequences used for alignment are histone H3 methyltransferase Clr4 from Schizosaccharomyces pombe (NP_595186.1), histone-lysine N-methyltransferase SUV39H1 isoform 2 from Homo sapiens (4507321:145-412), histone H3 methyltransferase DIM-5 from Neurospora crassa, (AAL35215.1), SET1 from Oryza sativa (AAK28975.1) putative histone-lysine N-methyltransferase from Phytophthora infestans (XP_002999311.1), and Helentrons from P. cambivora (AUVH01093707.1|: 299-12077), P. capsici (ADVJ01006715.1|:c14578-831), and P. infestans (see Additional file 8). [file 1759-8753-5-18-S10.pdf]

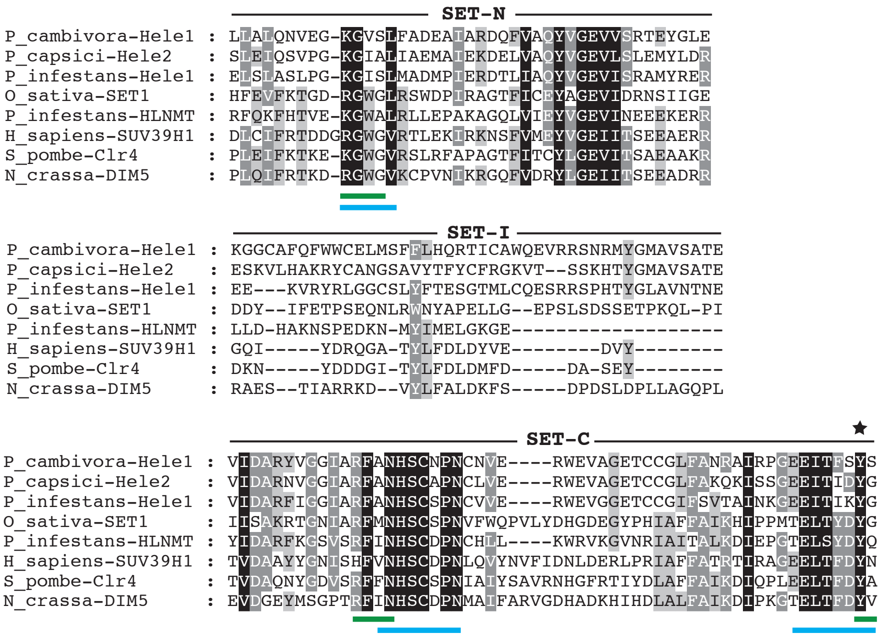
SET-N
P_cambivora-Hele1 : LLALQNVEG-KGVSLFADEAIARDQFVAQYVGEVVSRTEYGLE
P_capsici-Hele2 : SLEIQSVPG-KGIALIAEMAIEKDELVAQYVGEVLSLEMYLDR
P_infestans-Hele1 : ELSLASLPG-KGISLMADMPIERDTLIAQYVGEVISRAMYRER
O_sativa-SET1 : HFEVFKTGD-RGWGLRSWDPIRAGTFICEYAGEVIDRNSIIGE
P_infestans-HLNMT : RFQKFHTVE-KGWALRLLEPAKAGQLVIEYVGEVINEEEKERR
H_sapiens-SUV39H1 : DICIFRTDDGRGWGVRTLEKIRKNSFVMEYVGEIITSEEAERR
S_pombe-Clr4 : PLEIFKTKE-KGWGVRSLRFAPAGTFITCYLGEVITSAEAAKR
N_crassa-DIM5 : PLQIFRTKD-RGWGVKCPVNIKRGQFVDRYLGEIITSEEADRR
SET-I
P_cambivora-Hele1 : KGGCAFQFWWCELMSFFLHQRTICAWQEVRRSNRMYGMAVSATE
P_capsici-Hele2 : ESKVLHAKRYCANGSAVYTFYCFRGKVT--SSKHTYGMAVSATE
P_infestans-Hele1 : EE---KVRYRLGGCSLYFTESGTMLCQESRRSPHTYGLAVNTNE
O_sativa-SET1 : DDY--IFETPSEQNLRWNYAPELLG--EPSLSDSSETPKQL-PI
P_infestans-HLNMT : LLD-HAKNSPEDKN-MYIMELGKGE-------------------
H_sapiens-SUV39H1 : GQI-----YDRQGA-TYLFDLDYVE--------DVY--------
S_pombe-Clr4 : DKN-----YDDDGI-TYLFDLDMFD-----DA-SEY--------
N_crassa-DIM5 : RAES--TIARRKD--VYLFALDKFS-----DPDSLDPLLAGQPL
SET-C
P_cambivora-Hele1 : VIDARYVGGIARFANHSCNPNCNVE----RWEVAGETCCGLFANRAIRPGEEITFSYS
P_capsici-Hele2 : VIDARNVGGIARFANHSCAPNCLVE----RWEVAGETCCGLFAKQKISSGEEITIDYG
P_infestans-Hele1 : VIDARFIGGIARFANHSCSPNCVVE----RWEVGGETCCGIFSVTAINKGEEITIKYG
O_sativa-SET1 : IISAKRTGNIARFMNHSCSPNVFWQPVLYDHGDEGYPHIAFFAIKHIPPMTELTYDYG
P_infestans-HLNMT : YIDARFKGSVSRFINHSCDPNCHLL----KWRVKGVNRIAITALKDIEPGTELSYDYQ
H_sapiens-SUV39H1 : TVDAAYYGNISHFVNHSCDPNLQVYNVFIDNLDERLPRIAFFATRTIRAGEELTFDYN
S_pombe-Clr4 : TVDAQNYGDVSRFFNHSCSPNIAIYSAVRNHGFRTIYDLAFFAIKDIQPLEELTFDYA
N_crassa-DIM5 : EVDGEYMSGPTRFINHSCDPNMAIFARVGDHADKHIHDLALFAIKDIPKGTELTFDYV
